# Identification of a Hippocampus‐to‐Zona Incerta Projection involved in Motor Learning

**DOI:** 10.1002/advs.202307185

**Published:** 2024-07-03

**Authors:** Zhuo‐Hang Zhang, Bo Wang, Yan Peng, Ya‐Wei Xu, Chang‐Hong Li, Ya‐Lei Ning, Yan Zhao, Fa‐Bo Shan, Bo Zhang, Nan Yang, Jing Zhang, Xing Chen, Ren‐Ping Xiong, Yuan‐Guo Zhou, Ping Li

**Affiliations:** ^1^ The Molecular Biology Center State Key Laboratory of Trauma Burn and Combined Injury Department of Army Occupational Disease Daping Hospital Army Medical University (Third Military Medical University) 10 Changjiang Zhilu Chongqing 400042 China; ^2^ Department of Rehabilitation Medicine The Second Affiliated Hospital of Chongqing Medical University 76 Linjiang Road, Yuzhong Chongqing 400010 China

**Keywords:** acquisition, consolidation, hippocampus, motor learning, projections, retention/retrieval, zona incerta

## Abstract

Motor learning (ML), which plays a fundamental role in growth and physical rehabilitation, involves different stages of learning and memory processes through different brain regions. However, the neural mechanisms that underlie ML are not sufficiently understood. Here, a previously unreported neuronal projection from the dorsal hippocampus (dHPC) to the zona incerta (ZI) involved in the regulation of ML behaviors is identified. Using recombinant adeno‐associated virus, the projections to the ZI are surprisingly identified as originating from the dorsal dentate gyrus (DG) and CA1 subregions of the dHPC. Furthermore, projection‐specific chemogenetic and optogenetic manipulation reveals that the projections from the dorsal CA1 to the ZI play key roles in the acquisition and consolidation of ML behaviors, whereas the projections from the dorsal DG to the ZI mediate the retrieval/retention of ML behaviors. The results reveal new projections from the dorsal DG and dorsal CA1 to the ZI involved in the regulation of ML and provide insight into the stages over which this regulation occurs.

## Graphical abstract

The results reveal a previously unreported neuronal projection from dHPC to ZI involved in the regulation of ML behavior. The projections from the dorsal CA1 to the ZI play key roles in the acquisition and consolidation of ML behavior, whereas projections from the dorsal DG to the ZI mediate the retrieval/retention of ML behavior.

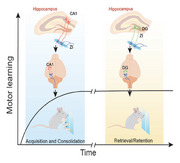

## 1 Introduction

Motor learning (ML), or motor skill learning, can be defined as a process that leads to relatively permanent changes in motor behavior through repeated interactions with the environment^[^ ^1^, ^2^ ^]^ and plays an important role in human rehabilitation and growth.^[^ ^3^, ^4^ ^]^ Motor learning and motor memory formation involve subprocesses similar to those of nonmotor memory formation: acquisition (or encoding), consolidation, and retention/retrieval.^[^ ^5^, ^6^ ^]^ Decades of research have explored the principles underlying these subprocesses in different learning processes,^[^ ^7^, ^8^, ^9^ ^]^ including error‐based learning, reinforcement learning, use‐dependent learning, and cognitive strategies,^[^ ^10^, ^11^ ^]^ all of which are crucial to daily life. However, many aspects of the neural and psychological mechanisms underlying motor learning remain poorly understood.

Multiple brain regions, including the primary motor cortex (M1), premotor cortex, supplementary motor area, cerebellar cortex, striatum, basal ganglia, and thalamus, are involved in motor learning.^[^ ^6^, ^12^, ^13^, ^14^ ^]^ The hippocampus (HPC) is a widely studied brain region thought to play an important role in higher cognitive functions such as learning, memory, and navigation.^[^ ^15^, ^16^ ^]^ Recent evidence has shown that the HPC is involved in the generation of ML behaviors and the overnight consolidation of motor memories.^[^ ^17^, ^18^, ^19^, ^20^ ^]^ In particular, aerobic exercise training may restore ML in Parkinson's disease (PD) patients and has a positive relationship with increased activity in the HPC, further indicating that the HPC is involved in ML.^[^ ^21^ ^]^ Although the neural responses underlying ML have been thoroughly investigated, various models have proposed that this process is supported by corticocerebellar, corticostriatal, and corticohippocampal networks.^[^ ^17^, ^18^, ^19^ ^]^ The dorsolateral prefrontal cortex plays an important role in the interaction between the hippocampal and striatal systems.^[^ ^18^, ^19^ ^]^ However, research on ways to modulate ML subprocesses (acquisition, consolidation, and retention/retrieval) via hippocampal‐targeted brain stimulation is lacking.

The zona incerta (ZI) directly controls various behaviors, including arousal, motivated behaviors, and motor posture.^[^ ^22^, ^23^ ^]^ In particular, it participates in the cognitive regulation of behavior, including directional changes in movements observed in turning, sniffing behaviors, attention, emotional stress‐induced aversive learning, and conditioned fear memory. This suggests that the ZI may be involved in regulating ML behaviors. Importantly, injury to the ZI can lead to abnormal movements (such as chorea),^[^ ^23^ ^]^ which is consistent with the symptoms of ML dyskinesia in PD patients, further suggesting that the ZI participates in the regulation of ML behaviors.

In the present study, we first identified projections from the dHPC (including projections from the dorsal dentate gyrus (DG) and CA1 subregions) to the ZI using adeno‐associated virus (AAV) and retro‐AAV labeling methods. We next revealed that the projections from the dorsal CA1 to the ZI play key roles in the acquisition and consolidation of ML behaviors, whereas projections from the dorsal DG to the ZI mediate the retrieval/retention of ML behaviors via a combination of optogenetics, chemogenetics, electrophysiology, and behavioral analysis. To our knowledge, these findings provide the first description of a neuronal projection from the dHPC to the ZI that is involved in the regulation of ML behaviors.

## 2 Results

### 2.1 A Novel Projection From The dHPC To The ZI

**Figure 1:**
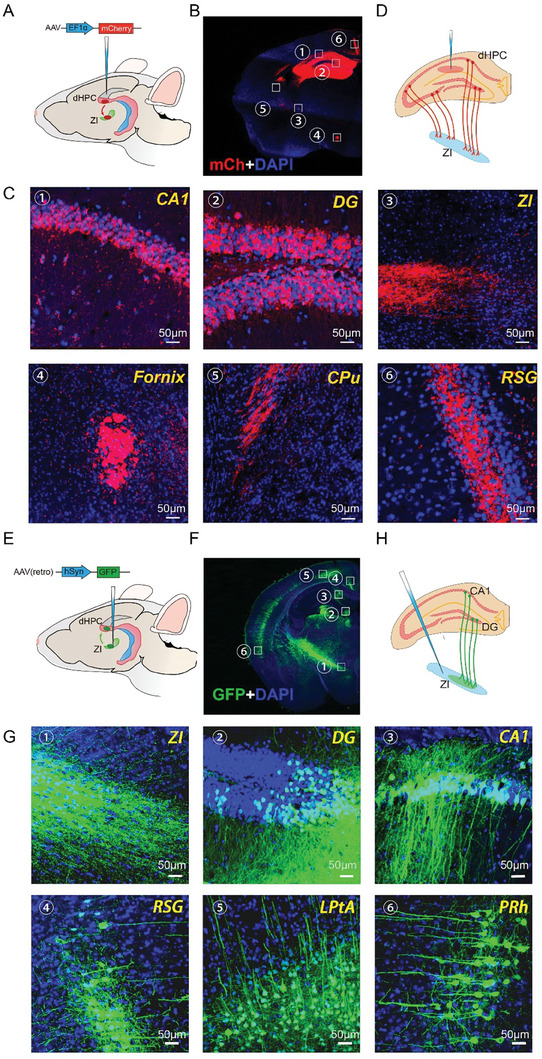
ZI neurons receive input from dHPC neurons. A) Schematic showing the viral labeling strategy using microinjections of AAV‐EF1α‐mCh into the dorsal hippocampus (dHPC) in mice. B) Representative fluorescence signal in the mouse brain after microinjection of AAV‐EF1α‐mCh, repeated in three mice. C) Higher magnification images of the sections in the white squares in (B). D) Schematic showing projection from the dHPC into the zona incerta (ZI). Green fluorescent‐labeled cell bodies can be observed in the DG and CA1, whereas green fluorescent‐labeled fibers can be seen in the ZI, fornix, CPu, and RGS. E) Schematic showing the design of retrograde labeling experiments using microinjection of AAV2‐retro‐hSyn‐eGFP into the ZI in mice. F) Representative fluorescence signals in mouse brains after microinjection of AAV2‐retro‐hSyn‐eGFP, repeated in three mice. G) Higher magnification images of the sections are shown in the white squares in (F). Green fluorescent‐labeled cell bodies are observed in the DG, CA1, RSG, LPtA, and PRh, whereas green fluorescent‐labeled fibers can be seen in the ZI. H) Schematic showing the projection from the DG and CA1 to the ZI. CPu, caudate putamen; RSG, granular retrosplenial cortex; LPtA, lateral parietal association cortex; PRh, perirhinal cortex.

To explore the projections from the dHPC using the AAV labeling method, a powerful means for establishing the organization and function of neural circuits,^[^ ^24^ ^]^ we microinjected AAV‐EF1α‐mCh into the dHPC of male wild‐type C57BL/6J mice (**Figure** **1**A); 1 month later, the mice were sacrificed. Histochemical analysis (Figure 1B,C) revealed red fluorescent‐labeled cell bodies in the dHPC, especially in the dorsal CA1 and DG; red fluorescent labeling in the fornix, the area through which the HPC outputs project^[^ ^25^ ^]^; and in the caudate putamen (CPu, striatum) and granular retrosplenial cortex (RSG), the output projection areas of the dHPC.^[^ ^26^ ^]^ More importantly, red fluorescent‐labeled fibers appeared in the ZI (Figure 1B,C). This suggested the existence of a projection from the dHPC to the ZI (Figure 1D). Additionally, we used AAV‐hSyn‐GFP following the same method as above, obtaining similar results (Figure S1A–C, Supporting Information) and further suggesting the existence of a projection from the dHPC to the ZI.

To further confirm the above findings, AAV2‐retro‐hSyn‐eGFP^[^ ^27^ ^]^ (which provides retrograde access to projecting neurons) was microinjected into the ZI (Figure 1E). We found that green fluorescent‐labeled cell bodies were expressed not only in various cortical subregions [such as the perirhinal cortex (PRh) and lateral parietal association cortex (LPtA)] and the central amygdala (Figure 1F,G; Figure S2A, Supporting Information)— the projection areas of the ZI^[^ ^28^, ^29^ ^]^—but also in the dorsal DG and CA1 (Figure 1F,G; Movies S1 and S2, Supporting Information). These results further confirmed the existence of a projection from the dHPC to the ZI and suggested that the dorsal DG and CA1 might also be output projection areas of the dHPC (Figure 1H). Moreover, green fluorescent‐labeled fiber terminals could also be seen in the ZI (Figure 1F,G), further indicating that the projections came from the dHPC. In addition, the green fluorescent‐labeled cells were colocalized with NeuN+ (red fluorescence, Figure S1D,E, Supporting Information), indicating that these cells were neurons.

### 2.2 Projections From The dHPC (dorsal DG and CA1) To The ZI

**Figure 2:**
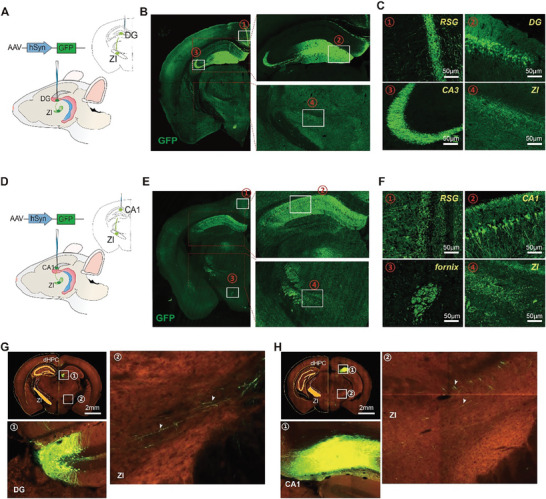
Projections of the dHPC to the ZI from the dorsal DG and CA1. A) Schematic showing the viral labeling strategy, which involved microinjection of AAV‐hSyn‐GFP into the dorsal DG of mice. B) Representative immunofluorescence images of GFP expression in the mouse brain (left), dHPC (right, upper), and ZI (right, lower) 1 month after microinjection of AAV‐hSyn‐GFP into the dorsal DG, repeated in three mice. C) Representative green fluorescent‐labeled cell bodies in the DG and representative green fluorescent‐labeled fibers in the RSG, CA3, and ZI. D) Schematic showing the viral labeling strategy, which involved microinjection of AAV‐hSyn‐GFP into the dorsal CA1 of mice. E) Representative immunofluorescence images of GFP expression in the mouse brain (left), dHPC (right, upper), and ZI (right, lower) 1 month after microinjection of AAV‐hSyn‐GFP into dorsal CA1, repeated in three mice. F) Representative green fluorescent‐labeled cell bodies in CA1 and representative green fluorescent‐labeled fibers in the RSG, fornix, and ZI. G) Panels from the Allen Reference Atlas. Representative immunofluorescence images of GFP expression in the mouse brain (left, upper), DG (left, lower), and ZI (right) after Cre‐expressing AAV tracing of axonal projections. H) Panels from the Allen Reference Atlas. Representative immunofluorescence images of GFP expression in mouse brain (left, upper) or CA1 (left, lower) and ZI (right) through Cre‐expressing AAV tracing of axonal projections. RSG, granular retrosplenial cortex.

To further explore whether the dorsal DG and CA1 participate in this projection, we microinjected AAV‐hSyn‐GFP into the dorsal DG and CA1 in mice (**Figure** **2**A,D, respectively). Green fluorescent‐labeled cell bodies were observed in the dorsal DG and CA1, and green fluorescent‐labeled fibers were found in the fornix, RSG, and ZI (Figure 2B,C,E,F; Figure S2B; Movie S3, Supporting Information). Additionally, green fluorescence‐labeled fibers were observed in the dorsal CA3 region in the dorsal DG microinjection group (Figure 2C), which reflects projections within subareas of the hippocampus.^[^ ^26^, ^30^ ^]^ These results demonstrated that the projections of the dHPC to the ZI come from the dorsal DG and CA1.

Furthermore, by searching the Allen Mouse Brain Connectivity Atlas (https://connectivity.brain‐map.org),^[^ ^31^ ^]^ we found green fluorescent‐labeled fibers in the ZI and green fluorescent‐labeled cells in the dorsal CA1 and DG after AAV microinjection into the CA1 and DG, respectively (Figure 2G,H). However, few green fluorescent filaments representing the projection end at the ZI were observed, which may be related to the limited number of neurons expressing fluorescence in the DG or CA1 (Figure 2G,H). Moreover, we did not observe green fluorescence signals in the ZI when AAV was microinjected into the dorsal CA3, dorsal CA2 (Figure S3A,B, Supporting Information), or ventral CA1 and CA3 (Figure S3C,D, Supporting Information). These results further confirmed that the projections of the dHPC to the ZI specifically originated from the dorsal CA1 and DG.

In addition, 1 month after microinjection of AAV2‐retro‐hSyn‐eGFP into the ZI, immunohistochemistry showed that green fluorescent‐labeled cells were extensively colocalized with NeuN+ (white arrow, Figure S4A, Supporting Information) and calmodulin‐dependent protein kinase II (CaMKII)+ cells (white arrow, Figure S4B, Supporting Information) and not with glutamic acid decarboxylase (GAD) (yellow arrow, Figure S4C, Supporting Information). The results demonstrated that the projections from the dHPC to the ZI were excitatory neurons, which is similar to the finding that the hippocampus sends excitatory projections to the nucleus accumbens (NAc).^[^ ^32^ ^]^

### 2.3 Optogenetic Inhibition of Projections From The dHPC To The ZI Impairs ML

**Figure 3:**
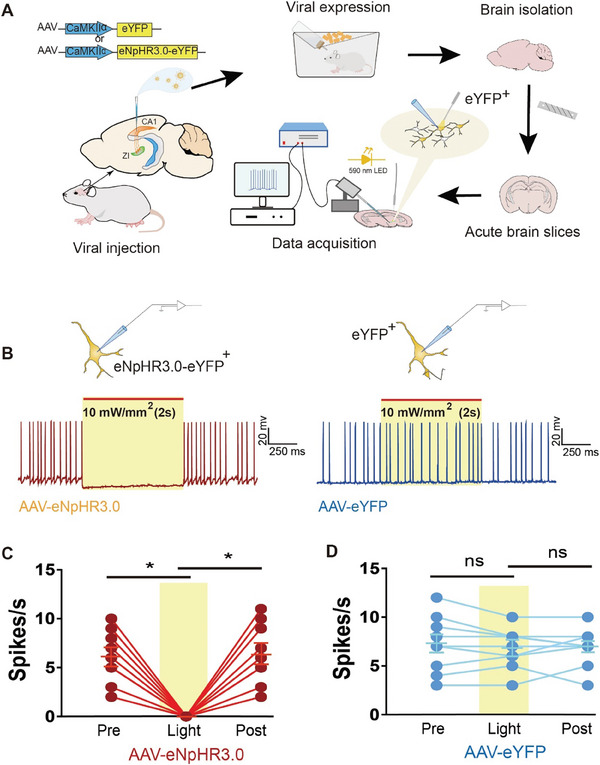
Functional validation of eNpHR3.0 in the HPC. A) Schematic showing the viral expression strategy involving microinjection of AAV‐CaMKIIα‐eNpHR3.0‐eYFP or AAV‐CaMKIIα‐eYFP into the dHPC in mice and whole‐cell patch‐clamp recording (ex vivo). B) A representative current‐clamped eYFP‐positive HPC neuron shows inhibition during light (10 mW mm^−2^ output, 2 s) activation of eNpHR3.0 (*left*), while no change is seen in eYFP controls (*right*) in brain slices. C) Quantification of the spike frequency of action potentials before, during, and after light stimulation in the eNpHR3.0 group (9 neurons from 5 mice, mean ± SD). One‐way ANOVA followed by the Bonferroni correction for post hoc testing. F_(2,16)_ = 36.66, *p* < 0.001 among groups; *p* < 0.001 for neurons pre‐ versus during light stimulation; *p* < 0.001 for neurons during versus post‐light stimulation; *p* = 0.999 for neurons pre‐ versus post‐light stimulation. D) Quantification of the spike frequency of action potentials before, during, and after light stimulation in eYFP controls (9 neurons from 4 mice). One‐way ANOVA was used, followed by Bonferroni‐corrected post hoc tests. F_(2,16)_ = 0.491, *p* = 0.591 among groups; *p* = 0.816 for neurons pre‐ versus during light stimulation; *p* = 0.999 for neurons during versus post‐light stimulation; *p* = 0.999 for neurons pre‐ versus post‐light stimulation. *p < 0.05 was considered significant. NS, no significance.

To investigate the role of the projections from the dHPC to the ZI in ML, we used optogenetic techniques, which provide the remarkable ability to assess the function of neural circuits.^[^ ^33^ ^]^ We first expressed the halorhodopsin (eNpHR3.0)‐eYFP fusion protein in the dHPC using an AAV vector under the control of the CaMKIIα promoter (AAV‐CaMKIIα‐eNpHR3.0‐eYFP) to target CaMKIIα‐positive excitatory cells (**Figure** **3**A). After 1 month of intra‐HPC microinjections of AAV‐CaMKIIα‐eNpHR3.0‐eYFP or eYFP alone (control), the mice were sacrificed, and brain slices containing hippocampal tissue were sectioned with a vibrating microtome. Similar to previous studies,^[^ ^34^, ^35^ ^]^ whole‐cell patch‐clamp recordings of the hippocampal slices showed that eNpHR3.0‐expressing neurons exhibited decreased firing rates during illumination (Figure 3B,C), whereas no change with illumination was observed in eYFP controls (Figure 3B,D); these findings confirmed the inhibitory effect of eNpHR3.0‐expressing neurons.

**Figure 4:**
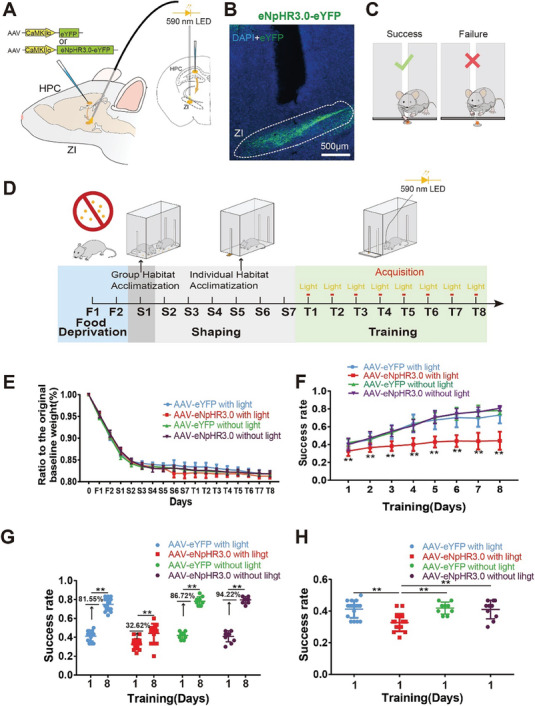
Optogenetic inhibition of projections from the dHPC to the ZI impairs ML. A) Schematic of the viral expression strategy and optogenetic approach for the inactivation of dHPC terminals in the ZI. B) Representative immunofluorescence images of eYFP‐expressing terminals (green) in the ZI and cannula above the terminal. C) Graphic representation of success and failure of mice in the single‐pellet reaching test. D) Experimental procedure. E) Body weight changed throughout all stages of the single‐pellet reaching test. Two‐way ANOVA was used, followed by Bonferroni's multiple comparisons test. F(3, 886) = 3.153, *p* = 0.024 for different groups (eYFP+light: *n =* 16 animals, eNpHR3.0+light: *n =* 16 animals, eYFP–light: *n =* 11 animals, eNpHR3.0–light: *n =* 10 animals), *p >* 0.05 for different groups at various time points. F) The success rate changed during the training phase. Two‐way ANOVA was used, followed by Bonferroni's multiple comparisons test. F(3, 368) = 258.10, *p <* 0.001 for different groups (eYFP+light: *n =* 16 animals, eNpHR3.0+light: *n =* 16 animals, eYFP–light: *n =* 11 animals, eNpHR3.0–light: *n =* 10 animals). *p* = 0.007, 0.005, <0.001, <0.001, <0.001, <0.001, <0.001, and <0.001 on Days 1–8, respectively, for eYFP+light versus eNpHR3.0+light. G) The success rate changed from day 1 to day 8 of the training phase in the different groups. Two‐tailed unpaired t‐tests were performed. *p <* 0.001 for day 1 versus day 8 in the eNpHR3.0+light group (*n =* 16 animals), *p <* 0.001 for day 1 versus day 8 in the eNpHR3.0–light group (*n =* 11), *p <*0.001 for day 1 versus day 8 in the eYFP+light group (*n =* 16), *p <*0.001 for day 1 versus day 8 in the eYFP–light group (*n =* 10). H) The success rate changed on day 1 of the training phase in the different groups. Two‐tailed unpaired t tests were performed (eYFP+light: *n =* 16 animals, eNpHR3.0+light: *n =* 16 animals, eYFP–light: *n =* 11 animals, eNpHR3.0–light: *n =* 10 animals). *p <* 0.001 for eYFP+light versus eNpHR3.0+light, *p <* 0.001 for eNpHR3.0+light versus eNpHR3.0–light, *p* = 0.001 for eYFP–light versus eNpHR3.0+light. The graph shows the mean ± SD. **p < 0.01 was considered significant.

We next tested the role of projections from the dHPC to the ZI in ML using a single‐pellet reaching task, which is not only used to study ML^[^ ^36^, ^37^ ^]^ in the hippocampus^[^ ^36^ ^]^ but can also be used in combination with optogenetics.^[^ ^38^ ^]^ One month after microinjections of either eNpHR3.0‐eYFP or eYFP alone (control) into the dHPC, the mice were surgically fitted with optical fibers at the ZI and allowed to recover for 1 week before being food‐restricted in preparation for behavioral testing (**Figure** **4**A,B). Figure 4C,D showed the experimental scheme, behavioral detection, and light stimulation (590 nm) applied simultaneously in the acquisition stage of the training phase (Figure 4D). During the whole behavioral experiment, there was no significant difference in weight change between the experimental (eNpHR3.0) and control (eYFP) mice at any time point during the test (Figure 4E). During days 0–8 of the training phase, the success rate of catching food significantly increased over time, plateauing in the eYFP with light (eYFP+light), eYFP without light (eYFP–light), and eNpHR3.0 without light (eNpHR3.0–light) groups but continuing to slowly increase in the eNpHR3.0 with light (eNpHR3.0+light) group (Figure 4F). Moreover, the success rate increased by 32.62% between days 1 and 8 in the eNpHR3.0+light group, a lower increase than that observed in the other groups (Figure 4G), indicating that inhibition of these projections impaired the ML process in these mice. In addition, the success rate of the eNpHR3.0+light group on the first day, equivalent to the retention/retrieval stage, relative to the first stage of learning in the training phase, was lower than that of the other groups (Figure 4H), indicating that inhibition of projections from the dHPC to the ZI impaired retention/retrieval in ML. Moreover, there was no significant difference between the eNpHR3.0+light group and the eYFP +light group in the rate of failure to touch and/or retrieve the pellet (Figure S5A, Supporting Information). Eshkol–Wachmann Movement Notation (EWMN) analysis, an assessment of digital dexterity,^[^ ^39^ ^]^ indicated that there was no significant difference between the eNpHR3.0+light group and the eYFP+light group in any of the 10 reaching components (Figure S5B, Supporting Information), suggesting that the activation of eNpHR3.0 had no impact on the digit dexterity of the mice.

**Figure 5:**
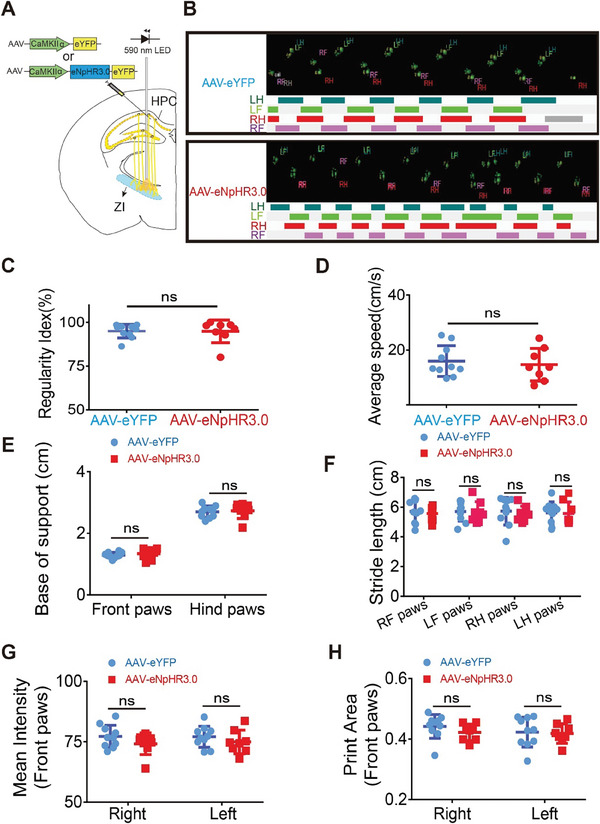
Optogenetic inhibition of projections from the dorsal HPC to the ZI had no effect on movement in catwalk gait analysis. A) Schematic of the viral expression strategy and optogenetic approach for inactivation of dorsal HPC terminals in the ZI. B) Schematic depicting mouse paw prints under the CatWalk system. Representative C) regularity index, D) average speed, E) base of support, G) mean intensity, and H) print area ∖ values in gait analysis for two groups receiving light stimulation to optogenetically inhibit projections from the dorsal HPC to the ZI. Two‐tailed unpaired *t*‐tests were used to compare the two groups (AAV‐eYFP: *n =* 10 animals versus AAV‐eNpHR3.0: *n =* 8 animals; C) *p* = 0.164, D) *p* = 0.850, *p* = 0.471 (E, front paws), *p* = 0.736 (E, hind paws), *p* = 0.661 (F, RF paws), *p* = 0.887 (F, LF paws), *p* = 0.635 (F, RH paws), *p* = 0.909 (F, LH paws), *p* = 0.182 (G, right), *p* = 0.336 (G, left), *p* = 0.247 (H, right) and *p* = 0.816 (H, left)) in terms of different behaviors. NS, no significance. RF: right fore, RH: right hind, LF: left fore, LH: left hind.

**Figure 6:**
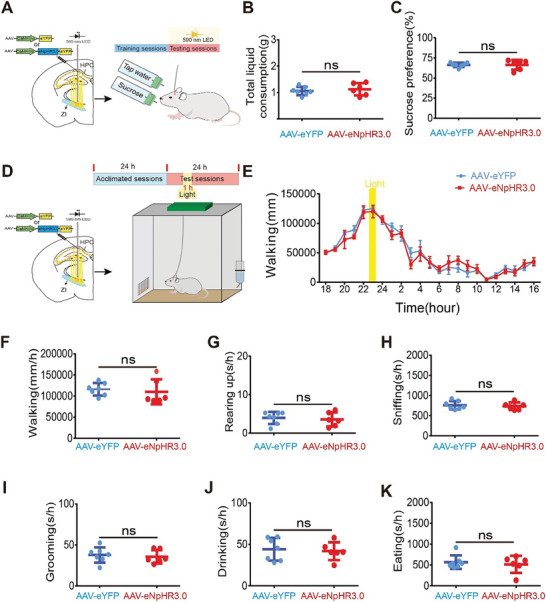
Optogenetic inhibition of projections from the dorsal HPC to the ZI had no effect on sucrose preference or movement in the HomeCageScan (HCS). A) Schematic drawing depicting the configuration of the sucrose preference test. B) Representative total liquid consumption values and C) sucrose preferences for the two groups stimulated with light to optogenetically inhibit projections from the dorsal HPC to the ZI. Two‐tailed unpaired t‐tests were used to compare the two groups (AAV‐eYFP: *n =* 10 animals versus AAV‐eNpHR3.0: *n =* 8 animals; B) *p* = 0.394 and C) *p* = 0.075). D) Schematic drawing depicting the configuration of the HCS. E) Movement distance per hour in the AAV‐eYFP and AAV‐eNpHR3.0 groups (AAV‐eYFP: *n =* 7 animals; AAV‐eNpHR3.0: *n =* 6 animals) during the test session was plotted. Two‐way ANOVA was used, followed by Bonferroni‐corrected post hoc tests. F (1, 253) = 0.3742, p = 0.541. *p >* 0.05 at different time points for AAV‐eYFP versus AAV‐eNpHR3.0. F) The amount of time spent per hour (s/h) walking, G) rearing up, H) sniffing, I) grooming, J) drinking, and K) eating in the HCS for the two groups stimulated with light to optogenetically inhibit projections from the dorsal HPC to the ZI was shown. Two‐tailed unpaired t‐tests were used to compare the two groups (AAV‐eYFP: *n =* 7 animals versus AAV‐eNpHR3.0: *n =* 6 animals; F) *p* = 0.608, G) *p* = 0.724, H) *p* = 0.798, I) *p* = 0.804, J) *p* = 0.622 and K) *p* = 0.608) in terms of the different behaviors. NS, no significance.

Additionally, to explore whether these projections were implicated in other behaviors, we continued to assess them for their potential roles in motor function, coordination, gait, rearing, and reward‐related behaviors. According to catwalk gait analysis (**Figure** **5**A,B), which is an updated behavioral assessment of motor function, coordination, and gait in mice,^[^ ^40^, ^41^ ^]^ the regularity index, average speed, base of support, stride length, mean intensity, and print area did not significantly differ between the eNpHR3.0 group and the eYFP group in the presence of light (Figure 5C–H). Furthermore, the sucrose preference test (SPT), which is frequently used to measure hedonic motivation and assess the rewarding and motivating qualities of food,^[^ ^42^, ^43^ ^]^ did not reveal any differences in total liquid or sucrose consumption between the eNpHR3.0 group and the eYFP group in the presence of light (**Figure** **6**A–C). Additionally, rodent behaviors in the home cage, including walking, rearing, grooming, and other activities, were classified and measured using HomeCageScan (HCS).^[^ ^44^ ^]^ Locomotor (walking), exploratory (rearing up and sniffing), grooming, eating, and drinking behaviors did not significantly differ between the eNpHR3.0 group and the eYFP group when exposed to light (Figure 6D–K). Collectively, these results suggested that the activation of eNpHR3.0 had no impact on these behaviors.

### 2.4 Elucidation of the Distinct Roles of Dorsal DG and CA1 in ZI Projections at Different Phases of ML with Optogenetic Inhibition

**Figure 7:**
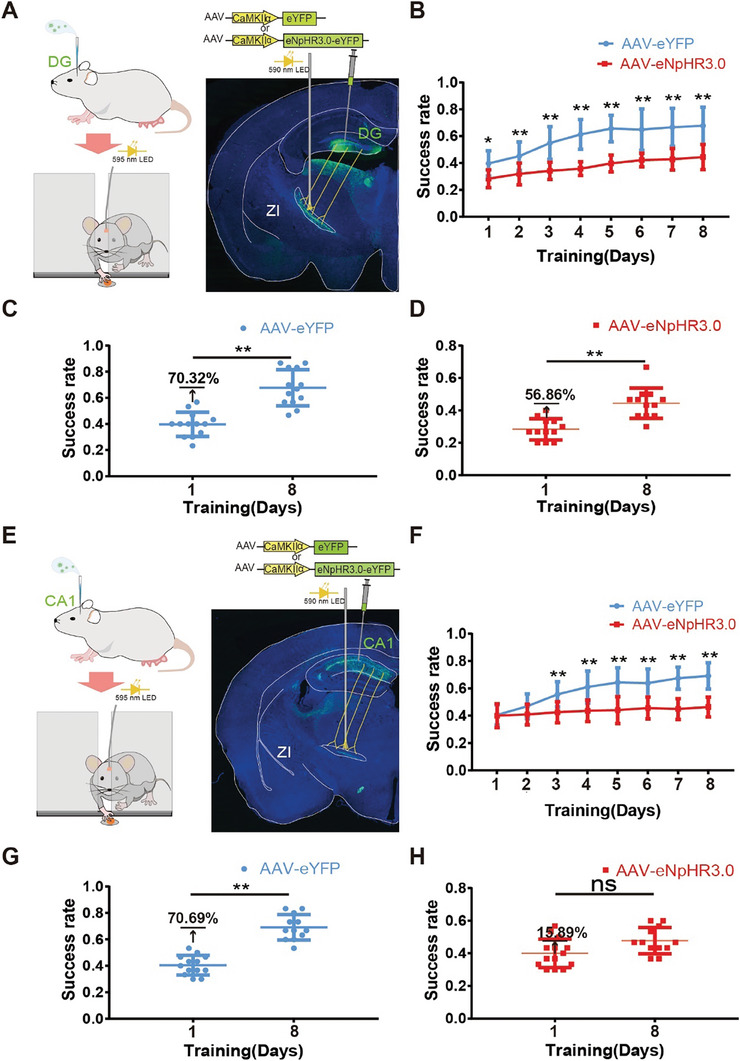
Distinct roles of the dorsal DG and CA1 projections to the ZI in the different phases of ML assessed by optogenetic inhibition. A) Schematic of the viral expression strategy, representative immunofluorescence images, and optogenetic approach for the inactivation of the terminals of the dorsal DG projections to the ZI. B) Changes in the success rate during the training phase following the optogenetic inhibition of projections from the dorsal DG to the ZI. Two‐way ANOVA was used, followed by Bonferroni's multiple comparisons test. F(1, 23) = 44.02, *p <* 0.001 for different groups (eYFP: *n =* 13 animals versus eNpHR3.0: *n =* 12 animals; *p* = 0.040, 0.010, <0.001, <0.001, <0.001, <0.001, <0.001, and <0.001 for Days 1–8, respectively). Changes in the success rate between Days 1 and 8 of the training phase in the C) eYFP group and D) eNpHR3.0 group. Two‐tailed unpaired *t*‐tests were used. *p <* 0.001 and *p <* 0.001 for day 1 versus day 8 in the eYFP and eNpHR3.0 groups, respectively (eYFP: *n =* 13 animals, eNpHR3.0: *n =* 12 animals). E) Schematic of the viral expression strategy, representative immunofluorescence images, and optogenetic approach for the inactivation of the terminals of the dorsal CA1 projections to the ZI. F) Changes in the success rate changes during the training phase following the optogenetic inhibition of projections from the dorsal CA1 to the ZI. Two‐way ANOVA was used, followed by Bonferroni's multiple comparisons test. F(1, 189) = 147.50, *p <* 0.001 for the different groups (eYFP: *n =* 13 animals versus eNpHR3.0: *n =* 14 animals; *p* = 0.999, 0.664, 0.002, <0.001, <0.001, <0.001, <0.001, and <0.001 at Days 1–8, respectively). Changes in the success rate between Days 1 and 8 of the training phase in the G) eYFP group and H) eNpHR3.0 group. Two‐tailed unpaired t‐tests were used. *p <* 0.001 and *p* = 0.057 for day 1 versus day 8 in the eYFP and eNpHR3.0 groups, respectively (eYFP: *n =* 13 animals, eNpHR3.0: *n =* 14 animals). The graph shows the mean ± SD. *p < 0.05 and **p < 0.01 were considered to indicate statistical significance. NS, no significance.

To further explore whether there are differences in the regulation of different phases of ML in the dorsal DG and CA1 projections to the ZI, we microinjected eNpHR3.0‐eYFP or eYFP alone into the dorsal DG and CA1 (**Figure** **7**A,E). The behavioral assessment and light stimulation methods described above were employed again here. Although the injection of eNpHR3.0 into projections from the dorsal DG to the ZI accompanied by light stimulation significantly reduced the success rate on the first day of training (Figure 7B,C), the success rate increased during the training stage, and by the eight‐day, it was significantly greater than that on the first day (Figure 7B,D). Although the success rate in the eNpHR3.0‐eYFP group did not reach that in the control DG‐ZI mice, the growth rates of the two groups were similar (Figure 7B,D), indicating that the acquisition of ML persisted. This result suggested that inhibition of the projections from the dorsal DG to the ZI inhibited the retention/retrieval of ML. However, the injection of eNpHR3.0 into the projecting cells from the dorsal CA1 to the ZI accompanied by light stimulation did not significantly influence the success rate on the first day of training (Figure 7F,G). The success rate of this group did not significantly increase during the training stage, so the success rate on the eighth day of training was not significantly greater than that on the first day (Figure 7F,H). This result not only suggested that the inhibition of projections from the dorsal CA1 to the ZI inhibited the acquisition of ML but also indicated that these projections play a key role in these processes. Although it was not easy to distinguish between retention/retrieval and consolidation because this task involves checking once a day, these results demonstrated that the dorsal DG and CA1 projections to the ZI play different roles during the different stages of ML.

Additionally, to explore whether these projections were involved in other behaviors, we continued to evaluate the behaviors that the hippocampus often participates in, including spatial memory and emotion. The spatial memory of the mice was examined by the Y‐maze test,^[^ ^45^ ^]^ while the anxiety‐like behaviors and locomotor activity of the mice were examined by the open‐field test.^[^ ^46^ ^]^ Through open‐field and Y‐maze tests, we found that there was no significant difference in the total distance traveled, time spent in the central arm, or time spent in the novel arm between the eNpHR3.0‐eYFP microinjection with light and eYFP microinjection with light groups in the dorsal DG or dorsal CA1 (Figure S6A–N, Supporting Information), which suggested that inhibition of these projections had no effect on locomotor activity, anxiety‐like behaviors or spatial memory. In addition, we performed additional behavioral tests after perturbing other hippocampal regions (specifically, the CA3 region of the hippocampus). We used AAV to transfect eNpHR3.0 into the CA3 region of the dorsal hippocampus (Figure S7A,B, Supporting Information) and demonstrated that the optogenetic inhibition of CA3 neurons via illumination following eNpHR3.0 transfection inhibited mouse spatial memory (Figure S7C,E, Supporting Information) but not motor or exploratory behaviors in the open field test (Figure S7F,H, Supporting Information). These behavioral analyses indicated that neurons in CA3 of the hippocampus could be optogenetically inactivated according to the role of the hippocampus in regulating spatial memory, consistent with previous reports on the role of the CA3 in spatial memory. Furthermore, inhibiting neurons in CA3 decreased the pellet‐reaching success rate of the mice, especially on days 7 and 8 of the training phase of the single‐pellet reaching test (Figure S7I–J, Supporting Information). This finding also indicates that optogenetic inhibition of CA3 could affect the acquisition/learning process. Hippocampal information processing occurs from the DG→CA3→CA2→CA1 to several regions, such as the cortex, amygdala, and ZI. Thus, optogenetic inactivation of CA3 neurons also showed effects similar to those seen when blocking the projections from CA1 to the ZI.

### 2.5 Chemogenetic Inhibition Further Confirmed the Distinct Roles of the Dorsal DG and CA1 Projections to the ZI in Different Stages of ML

**Figure 8:**
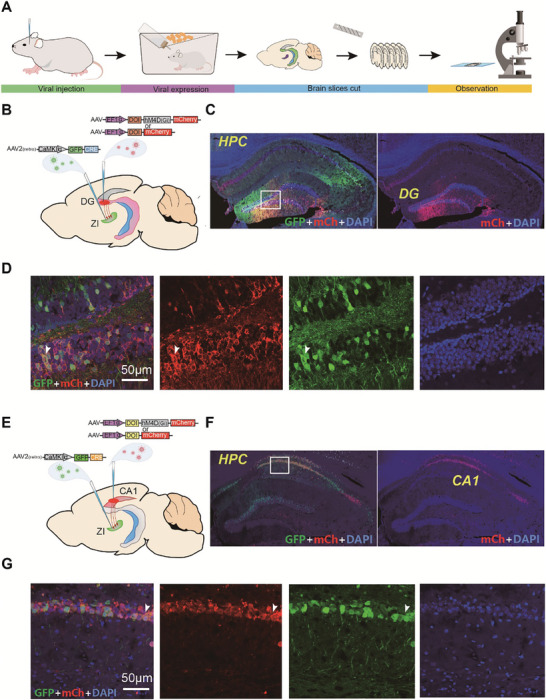
Expression of hM4D_(Gi)_ in projections from the dorsal DG or CA1 to the ZI. Schematic of the viral Cre/LoxP system strategy for determining the expression of hM4D_(Gi)_‐mCh A) in the neurons projecting from the dorsal DG to the ZI B) or from the dorsal CA1 to the ZI E) one month following microinjection of AAV‐EF1α‐DOI‐hM4D_(Gi)_‐mCh into the dorsal DG or into the dorsal CA1 and AAV2‐retro‐CaMKII‐GFP‐Cre into the ZI. Representative images of GFP‐expressing (green) and C) mCherry‐expressing (red) in the dorsal DG or F) CA1. Higher magnification images of the sections in the white squares in (D) and (G), respectively. The white arrow indicates the colocalization of green and red fluorescent‐labeled cells.

To further confirm the role of these projections in ML, especially in different stages, we adopted another behavioral test for ML: the rotarod test. Due to the need for long‐term regulation over the different stages of ML in the rotarod test, we used chemogenetic technology, which allows prolonged modulation of neuronal activity (for ≈12–24 h).^[^ ^23^, ^47^ ^]^ A Gi‐coupled inhibitory designer receptor exclusively activated only by designer drugs (DREADD),^[^ ^48^ ^]^ hM4D_(Gi)_, was used to inhibit the neuronal activity of the dorsal DG and CA1 projections to the ZI. Following microinjection of AAV‐EF1α‐DOI‐hM4D_(Gi)_‐mCh or AAV‐EF1α‐DOI‐mCh (control) into the dorsal DG or CA1 and AAV2‐retro‐CaMKII‐GFP‐Cre into the ZI (**Figure** **8**A,B,E), after one month, red fluorescent protein was expressed in the dorsal DG or CA1 and colocalized with green fluorescent protein representing AAV2‐retro‐CaMKII‐GFP‐Cre (white arrow; Figure 8C,D,F–G), indicating that hM4D_(Gi)_ was expressed in the dorsal DG or CA1 to ZI projections. Immunohistochemistry showed that both green and red fluorescent‐labeled cells were colocalized with purple (or grey) fluorescence (Figure S8A,B, Supporting Information), further suggesting that hM4D_(Gi)_ is expressed on the surface of neurons.

**Figure 9:**
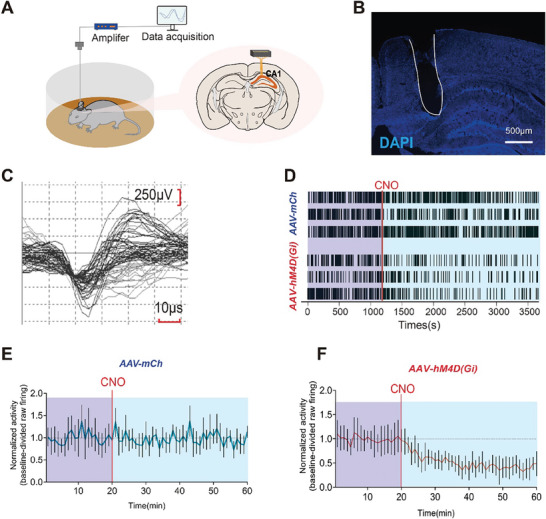
Functional validation of hM4D_(Gi)_ in the projections from the dorsal DG or CA1 to the ZI. A) Schematic showing freely behaving mice implanted with a head stage for electrophysiological recording. B) Representative immunofluorescence images of the electrodes above CA1. C) Representative waveform recorded from tetrodes in hM4D_(Gi)_‐expressing neurons projecting from the dorsal CA1 to the ZI one month after microinjection of Cre/LoxP AAV without CNO injection. D) Representative raster plot of spiking for 3 units in mCh or hM4D_(Gi)_‐expressing neurons after CNO injection. Baseline‐divided activity (mean ±SEM) in mCh(E) or hM4D_(Gi)_‐expression(F) neurons after CNO injection(*n =* 10).

We next addressed whether hM4D_(Gi)_ can inhibit neuronal activity in the absence of clozapine‐n‐oxide (CNO). We first used tetrode recording procedures before and after CNO injection (**Figure** **9**A,B). After a 20 min baseline recording session, the mice received an i.p. injection of 1 mg k^−1^g CNO. The average time from CNO injection to the onset of firing inhibition was 11.24 min (Figure 9C–F), similar to the findings of previous reports^[^ ^49^, ^50^ ^]^; the firing inhibition was consistent until 40 min after CNO injection (Figure 9C–F). During the 40 min post‐CNO‐injection period, activity was suppressed to 59% of baseline. Overall, these results demonstrated that CNO consistently quieted hM4D_(Gi)_‐expressing projection neurons after injection.

**Figure 10:**
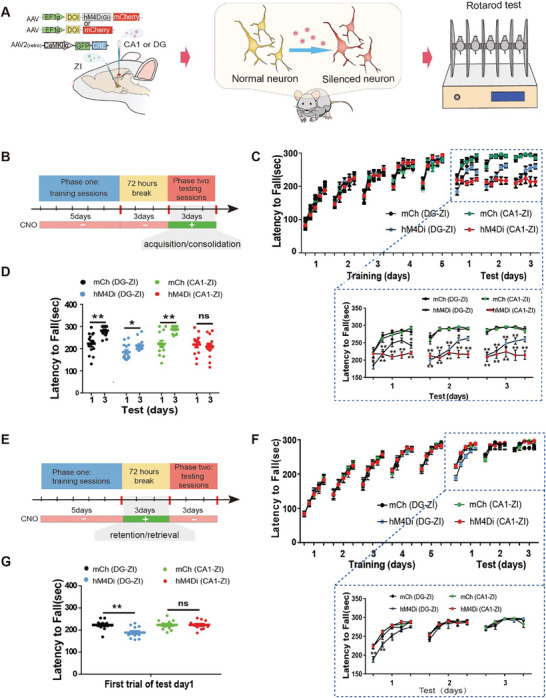
The distinct role of the dorsal DG and CA1 projections to the ZI at different phases of ML as assessed by chemogenetic inhibition. A) Experimental procedure. B) Diagram of the rotarod test protocol with CNO injection during the test session. C) Changes in the latency to fall during the rotarod test (B) for the different groups. Two‐way ANOVA was used, followed by Bonferroni's multiple comparisons test (*n =* 13 animals for each group). F(3, 240) = 37.02, *p <* 0.001 for the different groups on test day 1. *p* = 0.036, 0.001, 0.153, 0.187, and 0.024 for the 5 tests on day 1, for hM4Di (DG‐ZI) versus mCh (DG‐ZI), and *p <* 0.001, <0.001, <0.001, 0.048, and 0.118 for the 5 tests on day 1, respectively for hM4Di (CA1‐ZI) versus mCh (CA1‐ZI); F(3, 240) = 92.64, *p <* 0.001 for the different groups on test day 2. *p* = 0.036, 0.001, 0.153, 0.187, and 0.024 for the 5 tests on day 2 for hM4Di (DG‐ZI) versus mCh (DG‐ZI) and *p <*0.001 for the 5 tests on day 2 for hM4Di (CA1‐ZI) versus mCh (CA1‐ZI); F(3, 240) = 88.02, *p <* 0.001 for the different groups on test day 3. *p <*0.001, <0.001, 0.002, 0.008, and 0.111 for the 5 tests on day 3 for hM4Di (DG‐ZI) versus mCh (DG‐ZI) and *p <*0.001 for the 5 tests on day 3 for hM4Di (CA1‐ZI) versus mCh (CA1‐ZI). D) Changes in the latency to fall in the first trial between test days 1 and 3 for different groups. *p* = 0.001, *p* = 0.024, *p <* 0.001 and *p* = 0.646 for test day 1 versus test day 3 in the mCh (DG‐ZI) group, hM4Di (DG‐ZI) group, mCh (CA1‐ZI) group, and hM4Di (CA1‐ZI) group, respectively, according to two‐tailed unpaired t‐tests (*n =* 13 animals for the different groups). E) Diagram of the rotarod test protocol with CNO injection during the break session. F) Changes in the latency to fall during the rotarod test E) in the different groups. Two‐way ANOVA was used, followed by Bonferroni's multiple comparisons test (*n =* 12 animals for each group). F(3, 220) = 24.42, *p <* 0.001 for the different groups on test day 1. *p <* 0.001, 0.004, 0.032, 0.999, and 0.471 for the 5 tests on day 1 for hM4Di (DG‐ZI) versus mCh (DG‐ZI), and *p >* 0.05 for the 5 tests on test day 1 for hM4Di (CA1‐ZI) versus mCh (CA1‐ZI); F(3, 220) = 0.314, *p* = 0.815 for the different groups on test day 2. *p >* 0.05 for the 5 tests for hM4Di (DG‐ZI) versus mCh (DG‐ZI) and hM4Di (CA1‐ZI) versus mCh (CA1‐ZI) on test day 2; F(3, 220) = 0.226, *p* = 0.878 for the different groups on test day 3. *p >* 0.05 for the 5 tests for hM4Di (DG‐ZI) versus mCh (DG‐ZI) and hM4Di (CA1‐ZI) versus mCh (CA1‐ZI) on test day 3. G) Latency to fall in the different groups during the first trial on test day 1. *p* = 0.002 and *p* = 0.977 for mCh (DG‐ZI) versus hM4Di (DG‐ZI) and mCh (CA1‐ZI) versus hM4Di (CA1‐ZI), respectively, according to two‐tailed unpaired t‐test (*n =* 12 animals for different groups). The graph shows the mean ± SD. *p < 0.05 and **p < 0.01 were considered to indicate statistical significance. NS, no significance.

To further confirm the distinct roles of the dorsal DG and CA1 projections to the ZI at different stages of ML, we observed mouse ML behavior in the rotarod test after the inhibition of the neuronal activity of the dorsal DG and CA1 projections to the ZI by hM4D_(Gi)_ (**Figure** **10**A). Similar to previous experiments_,_^[^ ^51^, ^52^ ^]^ the experiment included a training phase (5 sessions), a 72 h break (retention/retrieval stage), and a test phase (3 sessions, acquisition, and consolidation stage), as shown in Figure 10B,E, to distinguish among the stages of ML. Because memory consolidation can occur between days during the test phase of the rotarod test^[^ ^53^ ^]^ and chemogenetics allows prolonged modulation of neuronal activity (for ≈12–24 h),^[^ ^23^, ^47^ ^]^ we merged acquisition and consolidation into one stage in our analysis. In the training phase, regarding the latency to fall, mCh(DG‐ZI) and mCh(CA1‐ZI) mice exhibited clear between‐session improvements that did not differ from those of the hM4Di(DG‐ZI) mice and the hM4Di(CA1‐Z1) mice (Figure 10C). When CNO was injected in the test phase, the latency to fall of the hM4Di(DG‐ZI), mCh(DG‐ZI), and mCh(CA1‐ZI) mice gradually increased in the subsequent trials (lower; Figure 10C), and the latency to fall in the first trial on the third day of the test phase was significantly longer than that on the first day (Figure 10D). However, the latency to fall of the hM4Di(CA1‐ZI) mice increased little in the following trials (lower; Figure 10C), and there was no significant difference in the latency to fall in the first trial on the third day and in the first trial on the first day (Figure 10D). Taken together, these results indicated that the CA1‐ZI projections participate in the acquisition and consolidation stage of ML and play a key role in these processes. In contrast, the DG‐ZI projections do not participate in these processes. Furthermore, the latency to fall in the first trial of the hM4Di(DG‐ZI) mice was significantly lower than that of the mCh(DG‐ZI) mice after CNO injection in the test phase, whereas there was no significant change in the latency to fall in the first trial of the hM4Di(CA1‐ZI) mice (Figure 10C,D). More importantly, a similar phenomenon was observed during CNO injection in the break phase (Figure 10F,G). These results indicate that DG‐ZI projections participate in the retention/retrieval stage of ML and that CA1‐ZI projections do not participate in these processes.

Additionally, in the open‐field, Y‐maze, and plus‐maze tests, we detected no significant difference in the total distance traveled, duration in the central arm, number of entries into the central arm, duration in the novel arm, number of novel arm entries, duration in the open arm, and number of open arm entries among the different groups at 30 min post‐CNO injection (Figure S9A–L, Supporting Information), which suggested that inhibition of these projections had no effect on locomotor, spatial‐memory or anxiety‐like behavior.

## 3 Discussion

The ability to acquire and refine motor behaviors is essential for maintaining functional independence throughout life. In recent years, the role of the HPC in ML has been extensively studied.^[^ ^17^, ^18^, ^19^, ^20^ ^]^ Several neuroimaging studies have identified the crucial role of the HPC in motor learning.^[^ ^54^, ^55^ ^]^ The HPC neural signatures described above are thought to support motor memory acquisition and predict successful motor memory retention.^[^ ^18^, ^55^, ^56^ ^]^ Moreover, in terms of neural mechanism research, studies in the last decade have shown that the corticohippocampal and corticostriatal networks play critical roles in ML.^[^ ^18^, ^57^ ^]^ It is not clear, however, whether other hippocampus‐associated networks are involved in ML.

In the current study, we identified projections from the dHPC to the ZI that have not previously been reported. The current findings were derived not only from the microinjection of AAV into the dHPC and of retro‐AAV into the ZI but also from data extracted from the Allen Mouse Brain Connectivity Atlas. Functional diversity arises from differences at multiple scales in the HPC, mainly including diverse afferent and efferent anatomic connections,^[^ ^26^, ^28^, ^29^, ^30^, ^58^ ^]^ including connections of the medial prefrontal cortex, orbital cortex, retrosplenial cortex (RSP), cingulate cortex, lateral septum (LS), and hypothalamus. Some of these connections were observed in the present study, enhancing the reliability of our research results. However, a drawback of the AAV microinjection approach is that the drug spreads from the site of infusion,^[^ ^59^ ^]^ leading to limited expression in the desired range. To address this limitation and identify direct outputs from specific cell populations in the dHPC, we used a dual‐AAV system (anterograde and retrograde viral tracers) combined with multiplex recombinase systems (Cre‐LoxP).^[^ ^59^ ^]^ Our findings further confirmed the existence of the dHPC to ZI projection in a cell type‐specific manner.

In addition, our microinjection experiments revealed that the neurons projecting to the ZI originate from the dorsal DG and CA1 in the dHPC. It is well known that CA1 is the primary output region from the HPC,^[^ ^60^ ^]^ which also supports the possibility of the existence of a projection from the dorsal CA1 to the ZI. Surprisingly, we first found that the dorsal DG projects to the ZI via excitatory neurons, indicating that the DG is also an output region of the HPC. Although no relevant reports were found, previous tracing studies proposed that somatostatin‐expressing interneurons in the DG project to the medial septum,^[^ ^61^, ^62^ ^]^ indicating that output projections from the DG are possible. Further study is needed to identify the specific type of neurons projecting from the dorsal DG to the ZI and other brain areas; nevertheless, our data not only suggest the novel discovery of projections from the HPC but also provide a new direction for research on the function of such projections.

More importantly, our study is the first to confirm that projections from the dHPC to the ZI are involved in ML behavioral regulation through two models according to optogenetic and chemogenetic manipulation. Many studies have shown that the HPC is involved in ML^[^ ^17^, ^18^, ^19^, ^20^ ^]^; however, direct evidence of the involvement of the ZI is lacking. The ZI is connected to nearly every neural center of the neuro‐axis, from the cerebral cortex to the spinal cord,^[^ ^29^ ^]^ and is involved in various functions,^[^ ^63^ ^]^ including locomotion, binge eating, sleeping, hunting, neuropathic pain, fear memory, and investigatory and seeking behavior. Adjustments of these behaviors are related to different input‒output associations. Therefore, considering the role of the hippocampus in ML, our findings establish a foundation by which future studies can investigate how the dHPC‐to‐ZI projections exert a regulatory effect on ML. In addition, as the main indicators in this study influence motor, spatial memory, and emotion functions, in our future experiments, we will explore whether other behaviors are regulated by dHPC‐to‐ZI projections.

Surprisingly, we also found that the projections to the ZI from the dorsal DG and CA1 regulate the retention (or retrieval) process and the acquisition and consolidation processes in ML, respectively. However, unlike for the role that the DG plays during memory encoding and consolidation, which is considered to be supported by strong evidence,^[^ ^64^, ^65^ ^]^ the role of the DG in memory retrieval is more controversial. Acute experimental DG inactivation impaired memory encoding but not recall in most studies,^[^ ^66^, ^67^ ^]^ whereas artificial reactivation of context‐encoding DG ensembles have been shown to trigger memory retrieval^[^ ^68^ ^]^ even after several weeks when the DG is not required for natural memory recall.^[^ ^69^, ^70^ ^]^ Exploring the role of DG subregions or different DG neuron types, including their synaptic inputs and outputs, may elucidate these discrepancies. Therefore, our study precisely regulated the projection pathway and revealed that the projections from the dorsal DG to the ZI may be involved in the regulation of the ML retrieval process. Similar situations also exist for CA1. For example, many studies have shown that CA1 is involved in the encoding, consolidation, or retrieval in hippocampal memory formation,^[^ ^65^, ^71^ ^]^ whereas some studies have reported that CA1‐lesioned rats are impaired in terms of memory retrieval but have no difficulty encoding new information.^[^ ^65^ ^]^ However, similar to our research on the neurons projecting from CA1 to the ZI, some researchers have found that a group of trace cells in the hippocampal CA1 subregion plays a key role in fear memory acquisition and consolidation.^[^ ^71^ ^]^ Therefore, our results not only show that different hippocampal subregions project to one brain region and play different regulatory roles but also indicate that studies on the functions of different brain regions should consolidate the functional characteristics of different DG neuron types and their synaptic inputs and outputs. In addition, the latency to fall and success rate in the rotarod test and the single‐pellet reaching task both increased similarly after the DG‐ZI projections were blocked but neither reached the values observed in the control group. Although this suggests that the DG‐ZI projections are not important in learning acquisition, more research is required to confirm this supposition.

The data obtained in this study have given rise to many other questions that should be considered for future exploration. For example, the differences in the regulatory effects between other projections regulating ML, such as the cortex‐to‐basal ganglia connection,^[^ ^72^ ^]^ and the projections of the HPC to the ZI remain unknown. Indeed, similar to M1, the HPC is crucial not only for movement execution but also for cognitive functions such as ML.^[^ ^73^, ^74^ ^]^ Recent behavioral studies have provided insights into the distinct learning processes underlying ML, with mechanisms mapped onto specific neural regions: error‐based learning (cerebellum), reinforcement learning (basal ganglia), cognitive strategies (prefrontal cortex), and use‐dependent learning (motor cortex).^[^ ^11^, ^75^ ^]^ Understanding the different neural mechanisms involved in motor performance and learning may inform novel interventions for enhancing motor skill learning. An important goal of future studies should be to explore whether there are neural projections from the DG to other brain regions and whether the pathways of these projections are shorter than that of the classical DG‐CA3‐CA1 circuit, which may play an important role in fast memory retrieval. The contribution of the HPC‐to‐ZI projection to other forms of memory is another important unknown. Whether the organizing principle revealed by these data applies to other memory systems is another intriguing question. In addition, ML has been shown to exhibit sex differences in mice and humans.^[^ ^76^, ^77^ ^]^ The investigation of such sex differences was beyond the scope of our study, as it was limited to male mice (see Experimental Section). However, future work could investigate whether there are sex differences in the regulation of ML behavior that originate from projections from the dorsal DG and CA1 subregions of the HPC to the ZI. In addition, optogenetic and chemical genetics techniques—which are frequently employed to investigate projection effects—based on the localization of viral injections into brain areas were employed in this work; however, there are still certain limitations to these techniques. First, the method for injecting viruses into specific brain regions may cause leakage to other regions, which could affect the specificity of the activity of the closed projections; second, the limited area of fiber optic illumination in optogenetics may affect the completeness of the closed projection. Nonetheless, to minimize the drawbacks of this approach, we attempted to optimize all aspects of the related experiment, including the determination of the injection site, dose, accuracy, precision, fiber optic selection, and the position and brightness of the light source.

## 4 Conclusion

In conclusion, we showed that a previously unreported neuronal projection from the dHPC to the ZI is involved in the regulation of ML behaviors. The projections from the dorsal CA1 to the ZI play key roles in the acquisition and consolidation of ML behaviors, whereas the projections from the dorsal DG to the ZI mediate the retrieval/retention of ML behavior. Although further studies are needed to explore the different roles of these projections in more specific stages of ML, these findings reveal for the first time projections from the dorsal DG and dorsal CA1 to the ZI and provide insight into their regulation of the different stages of ML.

## 5 Experimental Section

### Animals

Male wild‐type C57BL/6J mice (4–6 months old) were obtained from the Daping Hospital Animal Experimental Center. All mice were housed under a 12 L:12 D light/dark cycle in pathogen‐free conditions with ad libitum access to food and water. All animal procedures were approved by the Animal Ethics Committee of The Army Medical University, adhered to the Animal Research: Reporting of In Vivo Experiments (ARRIVE) guidelines, and were consistent with the National Institute of Health Guide for the Care and Use of Laboratory Animals. Studies have shown sex differences in the strength and types of ML behavior^[^ ^76^, ^77^ ^]^; in particular, fluctuations in estrogen levels in female subjects may affect behavior and ML.^[^ ^78^ ^]^ Therefore, including mice of both sexes in the study without differentiating between them would most likely have increased variability in the results, so it was decided to perform the initial study with male mice only.

### Vector Construction and AAV Preparation

All AAV viruses [AAV‐hSyn‐GFP, AAV‐EF1α‐mCherry, AAV‐CaMKIIa‐eYFP, AAV‐CaMKIIa‐eNpHR3.0‐eYFP, AAV‐EF1a‐DOI‐hM4D(Gi)‐mCherry, AAV‐EF1a‐DOI‐mCherry, and AAV‐CaMKIIa‐GFP‐2A‐CRE (retro)] were purchased from OBiO Technology (Shanghai) Corp. Upon purchase, the virus concentrations were ≈10^12^–10^13^ vector genomes (vg) mL^−1^; these were adjusted to 1.0 × 10^9^ vg mL^−1^ for stereotaxic injection via dilution with phosphate‐buffered saline (PBS).

### Stereotaxic Injection

Mice were anesthetized with 2% isoflurane via a nose cone, and their heads were fixed with a motorized mouse stereotaxic instrument (STOELTING model 51770). After applying a topical anesthetic (0.25% bupivacaine) and making a vertical incision in the skin over the skull, ≈1‐mm craniotomies were performed at a predetermined distance from the bifurcation of a major blood vessel. The coordinates used for locating the dHPC were as follows: 2.2 mm posterior to bregma, 1.5 mm from the midline, and 2.3 mm below the dura. The coordinates used for the dorsal DG were as follows: 2.2 mm posterior to bregma, 0.5 mm from the midline, and 2.3 mm below the dura. The coordinates used for dorsal CA1 were as follows: 2.2 mm posterior to bregma, 0.75 mm from the midline, and 1.7 mm below the dura. Finally, the coordinates used for the ZI were as follows: 2.2 mm posterior to bregma, 1.0 mm from the midline, and 4.2 mm below the dura. Viruses were injected using a glass micropipette attached to a 10 µL Hamilton syringe controlled by a Quintessential Stereotaxic Injector (STOELTING model 53311). A total of 0.5 µL (for the dHPC) or 0.2 µL (for other brain areas) of AAV was injected at a flow rate of 0.1 µL min^−1^ bilaterally for the behavioral experiments and on the left site for the tracing experiments. After injection, the needle was kept at the injection site for 10 min to allow sufficient absorption, then withdrawn slowly. After the surgery, the mice were allowed to recover and were returned to their home cages. One month later, the mice were perfused and processed for imaging or used for behavioral tests.

### Tissue Collection

Mice were sacrificed by exposure to CO_2_ and transcardial perfusion with 1× PBS followed by ice‐cold 4% paraformaldehyde. Mouse brains were collected and postfixed in 4% paraformaldehyde overnight and then dehydrated in PBS containing 30% sucrose for two days. Then, the dehydrated brains were stored in an ultralow temperature freezer (‐80 °C, MDF‐382ECN, SANYO).

### Immunohistochemistry

The fixed and dehydrated brains were frozen, coronally sectioned at 30 µm thickness on a sliding microtome (CM1950, Leica), and stored in a buffer containing 2% sodium azide at 4 °C until staining. The sections were blocked for 1 h in 0.1% Triton X‐100 in PBS and 10% goat serum and then incubated with primary antibody diluted in blocking buffer overnight at 4 °C. After staining, the sections were brought from 4 °C to room temperature, rinsed 3× with PBS for 5 min, and incubated with fluorophore‐conjugated secondary antibodies for 2 h at room temperature. Then, the sections were rinsed 3× with PBS for 5 min. After incubation with DAPI for 5 min at room temperature, the sections were rinsed 3× with PBS for 5 min again. Finally, the sections were mounted onto glass slides. For the tracing experiments, the sections were directly mounted or incubated with DAPI for 5 min and then mounted. The sections were analyzed, and images were acquired under a confocal laser scanning microscope (TCS SP8, Leica Microsystems, Germany). The primary antibodies used included mouse anti‐NeuN (Abcam, cat. no. ab104224, 1:500), rabbit anti‐CaMKII (Abcam, cat. no. ab52476, 1:200), and mouse anti‐GAD antibody (Abcam, cat. no. ab26116, 1:100). The secondary antibodies used were goat anti‐rabbit (Cy3, Abcam, cat. no. ab6939, 1:500), goat anti‐mouse (Cy3, Abcam, cat. no. ab97035, 1:500) and goat anti‐mouse antibody (Alexa Fluor 647, Abcam, cat. no. ab150115, 1:500).

### Allen Mouse Brain Atlas

The Allen Mouse Brain Atlas (https://connectivity.brain‐map.org) offers the Adult Mouse Connectivity Atlas, an image database of axonal projections labeled by viral (AAV) tracers and visualized using serial two‐photon tomography. Among the tools included in this resource, the injection site (filter source structures: hippocampus) was used to search for projections associated with the structures of interest. Image Viewer was used to browse the experiments via 3D thumbnails and pan through the 140 coronal slices of each experiment. The mouse strains in the atlas include wild‐type and transgenic Cre lines. Adobe Photoshop (ESTK version 3.8) was used for photo editing, and Adobe Illustrator (version 16.0) was used to generate the figures.

### Optogenetics and Whole‐Cell Patch‐Clamp Recording (Ex Vivo)

The mice received intra‐HPC microinjections of viral vectors carrying either eNpHR3.0‐eYFP or eYFP alone (control) with the CaMKII promoter. After 1 month, the mice were deeply anesthetized with isoflurane and decapitated. The brain was removed within 60 s and bathed in an ice‐cold solution consisting of 210 mm sucrose, 3.0  mm KCl, 0.75  mm CaCl_2_, 3.0  mm MgSO_4_, 1.0  mm NaH_2_PO_4_, 26  mm NaHCO_3_, and 10  mm glucose, saturated with 95% O_2_ and 5% CO_2_. Hippocampal slices were cut coronally at a thickness of 320–350 µm using a VT‐1200s vibratome (Leica), a movement velocity of 0.06 mm ^−1^s, and an oscillation amplitude of 2 mm. The slices were kept in artificial cerebrospinal fluid (ACSF) consisting of 125  mm NaCl, 2.5  mm KCl, 25  mm NaHCO_3_, 1.25  mm NaH_2_PO_4_, 1.2  mm MgCl_2_, and 20  mm glucose, saturated with 95% O_2_ and 5% CO_2_, for 45–60 min at 34 °C. The slices were allowed to recover for at least 1 h before recording. During recording, a single brain slice was transferred to a chamber and submerged in ACSF flowing at a rate of 2–3 mL min^−1^. Patch pipettes were filled with an intracellular solution of 105  mm K‐gluconate, 30  mm KCl, 4  mm Mg‐ATP, 0.3  mm Na‐GTP, 0.3  mm EGTA, 10  mm HEPES, and 10  mm Na‐phosphocreatine at a pH 7.35.

Picrotoxin (50 µm) and CNQX (10 µm) were used throughout the experiment to block inhibitory and excitatory synaptic transmission, respectively. The cells were visualized using a Scientifica sliceScope Microscope (Plexon, Inc.). Whole‐cell current signals were recorded with a MultiClamp 700B (Axon), Clampex 10, and Clampfit 10.6 software (Molecular Devices). Current clamp recordings were filtered at 10 kHz and digitized at 40 kHz. The pipettes were fabricated from borosilicate glass (BF100‐58‐10, Sutter Instrument) to a resistance of 6–8 MΩ. After the establishment of the whole‐cell configuration and equilibration of the intracellular pipette solution with the cytoplasm, the light intensity was adjusted to obtain a maximal response without overstimulation. Green fluorescent‐labeled cells were targeted by green‐filtered white LED illumination (pE‐300, CoolLed). Optogenetic stimulation was delivered using a red‐filtered white LED (pE‐300, CoolLed) controlled by a digital‐analog converter (Digidata 1550B, Axon) at an intensity of 10 mW mm^−2^ for 2 s and passed through a ×40 objective positioned above the recorded neuron.

### Chemogenetics and Electrode Recording in Awake Mice

Mice that received microinjections of AAV‐EF1α‐DOI‐hM4D_(Gi)_‐mCh into the dorsal CA1 and AAV‐CaMKIIa‐GFP‐2A‐CRE (retro) into the ZI for one month were anesthetized with 2% isoflurane via a nose cone and placed in a stereotaxic frame. The scalp was sterilized with 0.1% iodine, and a midline incision was made. A craniotomy ≈2 mm in diameter (for electrode bundles) was made by drilling the skull 2.2 mm posterior to the bregma and 1.5 mm from the midline. Three holes for placing anchoring screws were drilled around the electrode bundle hole. A 16‐channel microwire array (KD‐MWA, Kedou Brain‐Computer Technology) consisting of 4×4 polyimide‐coated tungsten wires (35 µm diameter, 200 µm pitch, and row spacing) was unilaterally implanted in the left hemisphere through the electrode bundle hole (2.0 mm below the dura). Silver wire was soldered to a stainless‐steel screw inserted into the hole above the cerebellum for grounding. The electrodes were cemented to the skull with the anchoring screws with dental acrylic. The mice were then allowed to recover for at least one week. Before recording, the implanted microwire array was connected to a Plexon multichannel acquisition processor. Neural waveform sorting was performed using the OmniPlex Neural Recording Data Acquisition System (Plexon, Inc.). Electrical signals were amplified 100–1000 times, sampled at 32 kHz, and filtered at 600–6000 Hz; acquired data were analyzed by using the commercially available software Offline Sorter (Plexon Inc.).

### In Vivo Optogenetic Stimulation—Implantation of the Optical Fiber Cannula

A fiberoptic cannula was made from an optical fiber 200 µm in diameter attached to a cannula (CFMLC12L10, Thorlabs). One month after AAV‐CaMKIIa‐eNpHR3.0‐eYFP or AAV‐CaMKIIa‐eYFP microinjection into the dorsal CA1 or DG, the mice were anesthetized, and the fiberoptic cannula was stereotactically implanted into the brain above the ZI (2.2 mm posterior to bregma, ±1.0 mm from the midline (bilateral), 4.2 mm below the dura). The cannula was secured to the skull with cold‐curing denture repair material (XH383P04, vertex‐Dental B.V.). After the implantation surgery was completed, the mice were returned to their individual cages and allowed to recover for 7 days before the behavioral tests.

### In Vivo Optogenetic Stimulation—Light Stimulation

The mice were habituated to body restraint, and their cannulae were connected to optical fiber sleeves (ADAL1, Thorlabs) once a day for three days before behavioral testing. For optical stimulation, the light was delivered using a PlexBright optogenetic stimulation system (Plexon Inc.) consisting of a four‐channel LED driver (OPT/4LED, Plexon Inc.) through a 1 × 2 rotary joint splitter (OPT/COM‐Dual, Plexon Inc.) and constant yellow LEDs for inhibition (compact LED, 590 nm, Plexon Inc.) during behavioral test. The output at the end of each patchcord (200/230 µm, 0.5NA, Plexon Inc.) was ≈5 mW mm^−2^.

### In Vivo Optogenetic Stimulation—Light Administration

Before optogenetic stimulation, the mice were allowed to acclimate for 30 min in the test room; then, the optical fiber was connected, and the mouse was allowed an additional 5 min for habituation. The mice received bilateral optogenetic stimulation when first placed in the test site for the open‐field test, plus‐maze test, and gait analysis. However, bilateral optogenetic illumination was delivered when the mice were first placed into the test site in the training phase, in the second trial of the test phase, and in the testing period in the single‐pellet reaching test, Y‐maze test, and sucrose preference test, respectively.

### Stereotaxic Surgery for DREADD Delivery

The animal surgery and virus injection methods were the same as those described above. Before electrode recording or behavioral testing, the mice received microinjections of AAV‐EF1α‐DOI‐hM4D_(Gi)_‐mCh or AAV‐EF1α‐DOI‐mCh (control) into the dorsal CA1 or dorsal DG and of AAV‐CaMKIIa‐GFP‐2A‐CRE (retro) into the ZI for one month.

### CNO Administration

For electrode recording, after a 20 min baseline recording, the mice received an i.p. injection of 1 mg k^−1^g CNO (Tocris Bioscience) dissolved in 0.9% saline and were immediately returned to the chamber for another 40 min of recording. Before behavioral testing, the mice were first intraperitoneally injected with 1 mg k^−1^g CNO dissolved in 0.9% saline for 30 min in the test room.

### Behavioral Tests—Single‐Pellet Reaching Test

The single‐pellet reaching test was performed as previously described.^[^ ^36^ ^]^ Assessment of motor learning was carried out using a clear Plexiglas training chamber (20 cm × 15 cm × 8.5 cm) with 3 vertical slits (0.5 cm wide and 13 cm tall, 1 slit on the “shaping” edge, and 2 slits on the “training” edge) on the front wall. The mice were able to reach the food on the divot slots of the food platform through the slits. The mice were food‐restricted until they reached 85–90% of their initial body weight. Weight was monitored daily throughout the experiment. In the food deprivation phase, each mouse was first weighed to obtain a baseline body weight and then subjected to food restriction for 2 days. They were given 0.1 g of food per 1 g of body weight per day. The methods used for the shaping phase (days 3–7) were as follows. On day 1, two mice were simultaneously placed in one training chamber containing ≈20 seeds per mouse for consumption. The mice were kept in this training chamber for 20 min and then returned to their home cage. On day 2, the setup was the same as that on day 1, but only one mouse was placed in the training chamber. On day 3, the forelimb dominance of the mice was determined by allowing them to reach for food outside the chamber through the slit; typically, the mice performed 70% of the reaches with the same forelimb. In the training phase (days 8+), the mice were individually placed in the chamber with the double‐slit side facing downward, and the pellets were placed on the food platform in the divot corresponding to the preferred paw. The mice were trained to reach for the food with their preferred paw for 30 attempts or 20 min per day; after the training was completed, the mice were returned to their home cages. The mouse reaching behavior was categorized as follows: 1) success: the mouse reached the seed with the preferred paw, grasped the seed, and retrieved it for feeding, putting it in its mouth; 2) drop: the mouse reached for and grasped the seed with the preferred paw but dropped the seed before putting it into its mouth; or 3) failure: the mouse reached toward the seed with the preferred paw but missed or knocked the seed from the platform. The success rate (%) was calculated as the number of successful reaches out of the total number of reaching attempts. EWMN analysis was employed on days 1 and 8 of the training phase, and same the rating scales employed in a previous study^[^ ^39^ ^]^ were used here to rate the movements. Each movement was rated on a 3‐point scale, with 0 representing no movement, 1 representing the occurrence of a movement with some ambiguity or the movement being present but not finished, and 2 representing the absence of movement. Before the start of the test, the mice were allowed to acclimate for 30 min in the test room; the optical fiber was then connected, and the mouse was allowed to adapt for an additional 5 min. The methods for optogenetically stimulating the mice during the acquisition stage of the learning phase were described above.

### Behavioral tests—Sucrose preference Test

Using a two‐bottle choice paradigm, the preference for a mild sucrose solution (1% wt/vol, Solarbio, Beijing, China) over the water was evaluated as previously described.^[^ ^79^ ^]^ Briefly, prior to the behavioral test, mice implanted with optical fiber cannulae were trained in operant chambers with two bottles and a house light (40 lux). One bottle contained tap water, whereas the other contained tap water with 1% sucrose. The food was removed, and the mice were presented with two bottles containing either plain water or sucrose solution after 4 hours of water deprivation. To calculate the amount of liquid consumed, the bottle was weighed before and after the experiment. By dividing the amount of liquid sucrose ingested during the 1‐hour test period by the total amount of water consumed, the sucrose preference of the mouse was determined. If the bottles leaked during the test, the corresponding data were omitted. The protocol for mouse optogenetic stimulation was described above.

### Behavioral Tests—Gait Analysis

Gait analysis was performed as previously described^[^ ^40^, ^41^ ^]^ with the video‐based digital gait analysis system CatWalk XT (Noldus Information Technology, Wageningen, Netherlands). Records for each mouse were collected only when all four paws completed at least three passages through the glass runway. Briefly, the videos were analyzed using CatWalk XT software (Noldus Information Technology), and footprints were automatically or manually labeled as right fore (RF), right hind (RH), left fore (LF), or left hind (LH) paws. Data such as the regularity index, average speed, base of support, stride length, mean intensity, and print area were all automatically examined and exported once each individual footprint was identified. An experimenter who was blinded to the group composition conducted the gait analysis test. The protocol for mouse optogenetic stimulation was as described above.

### Behavioral Tests—HomeCageScan

HCS was performed as previously described.^[^ ^80^ ^]^ Briefly, all animals with implanted optical fiber cannulae spent 24 hours adapting to PhenoTyper 4500 cages in the Home Cage Rack (Noldus Information Technology, Wageningen, The Netherlands) before the test sessions, which took place from 18:00–18:00. Subsequently, data on behaviors including walking, rearing up and sniffing, grooming, eating and drinking behaviors were collected by EthoVision XT 10.0 video‐based tracking software (Noldus Information Technology, Wageningen, The Netherlands) for 23 hours in the test sessions. The mice were subjected to optogenetic stimulation for 1 hour when the time reached the sixth hour, the time at which they were most active. The protocol for mouse optogenetic stimulation was as described above.

### Behavioral Tests—Rotarod Test

A computer‐controlled rotarod apparatus (SA102, SANS, Jiangsu Sionsi Biotechnology Co., Ltd.) was used to conduct the rotarod test; this apparatus consists of six 3 cm‐diameter drums, the surfaces of which were covered with hard chloroethylene, which prevented the mice to grip the surface. The rotarod was set to accelerate from 4 to 40 revolutions per minute over 300 s, and the latency to fall from the rotarod (latency to fall) was recorded. the mice performed 5 consecutive trials per session and 1 session per day. The maximum trial time was 5 min. The rest period between trials was ≈30 s. After each trial, the apparatus was cleaned with 70% ethanol for the next use. Before each daily session, the mice were allowed to acclimate for 30 min in the test room and were placed on a stationary drum for 3 min for habituation. Latency was recorded as the duration from the start of the trial until the mouse fell off the rotarod. The CNO administration protocol for the mice used in the acquisition stage of the training phase was as described above.

### Behavioral tests—Open‐Field Test

The open‐field chamber (50 cm × 50 cm× 45 cm) consists of an arena enclosed by four white PVC walls with a grid beam. The mice were transferred from their cage by the tail and placed in the center of the open‐field area. The five‐minute test was initiated when the mice began to move and broke the grid beam. The data were collected using Noldus Ethovision XT 12.1 software (Noldus Information Technology, Wageningen, The Netherlands). The center area was defined as the 25 × 25 cm central section of the chamber. The total distance traveled was used to evaluate locomotor activity, and the duration in the center area was used to estimate anxiety levels in an open environment, as commonly used in this paradigm. The protocol for mouse optogenetic stimulation and CNO administration was as described above.

### Behavioral Tests—Y‐Maze

The Y‐maze consists of 3 arms, a starting arm (always open), a second arm (always open), and a novel arm (blocked in the first trial and open in the second trial), which were enclosed with 15 cm tall walls. The angle between each arm was 120°. The two trials of a single Y‐maze test were separated by a 2‐hour interval. The first training trial lasted for 10 min; in this trial, the animals were allowed to explore the starting arm and the second arm freely, while the novel arm was blocked. After a 2‐hour interval, the second trial was conducted by placing the mouse in the starting arm and allowing it to explore all three arms for 5 min. The movement of the mice in the 3 arms was recorded by a digital video camera placed above the apparatus, and the data were collected using Noldus Ethovision XT 12.1 software (Noldus Information Technology, Wageningen, The Netherlands). The protocol for mouse optogenetic stimulation and CNO administration was as described above.

### Behavioral Tests—Elevated Plus Maze

The elevated plus maze consists of 4 arms (2 open arms and 2 closed arms); the closed arms were enclosed with 15‐cm walls on three sides. The entire maze was elevated 1 m above the floor. The mice were allowed to acclimate for 10 min in the test room prior to the start of the test. During the test, the mice were placed in the center of the elevated plus maze facing the same open arm. The mice were allowed to explore freely for 5 min while being recorded by a digital video camera placed above the apparatus. The data were collected using Noldus Ethovision XT 12.1 software (Noldus Information Technology, Wageningen, The Netherlands). The protocol for mouse optogenetic stimulation and CNO administration was as described above.

### Statistical Analysis

All results were presented as the mean ± standard deviation (SD) and were analyzed in GraphPad Prism 7.0 (GraphPad Software, CA). All statistical analyses were two‐tailed. Shapiro‐Wilk normality test was used to assess normal distribution. Grubbs’ test was used to identify and remove outliers. No statistical methods were used to predetermine the necessary sample size, but the sample sizes were similar to those reported in previous publications.^[^ ^81^, ^82^ ^]^ Comparisons among groups were performed with a two‐tailed t‐test or one‐ or two‐way repeated‐measures analysis of variance (ANOVA), as appropriate. Bonferroni's multiple comparisons test was performed to clarify the sources of all main effects. *p <* 0.05 was considered to indicate statistical significance (*p < 0.05, **p < 0.01). For all experiments, including animal behavioral tests, mice were excluded if health concerns arose, such as infection, bleeding, or significant changes in body weight. The mice were also excluded if GFP or mCherry was not detected specifically in the dorsal DG or CA1. All experiments were replicated multiple times with independent animals, and the same experiments were repeated independently by 2 experimenters at different times. Animals in the same litter were randomly assigned to different treatment groups, and the experimenters were blinded to the treatment group. Injection sites and viral expression were confirmed for all animals. Mice with incorrect injection sites or optic fiber placement were excluded from the data analysis.

## Conflict of Interest

The authors declare no conflict of interest.

## Author Contributions

Z.‐H.Z. and B.W. contributed equally to this work. P.L. conceptualized and designed the experiment. Z.‐H.Z., B.W., and P.L. conducted the experiments and analyzed data. Y.P., Y.‐W.X., and C.‐H.L. helped with virus injection. Y.‐L.N. and Y.Z. helped with vector construction. Y.P., F.‐B. S., B.W., and N.Y. helped with immunohistochemical experiments. C.‐H.L., J.Z., B.Z., and X.C. helped with the behavior test. Y.‐W.X. and C.‐H.L. helped with optogenetic experiments. R.‐P.X. helped with electrophysiological experiments. Y.‐G. Z. helped with data analysis. The manuscript was written by Z.‐H.Z., B.W., and P.L. P.L. supervised all phases of the project.

## Supporting information

Supporting Information

Supplemental Movie 1

Supplemental Movie 2

Supplemental Movie 3

## Data Availability

The data that support the findings of this study are available on request from the corresponding author. The data are not publicly available due to privacy or ethical restrictions.
