# Supplementary material for: Identification of a Hippocampus‐to‐Zona Incerta Projection involved in Motor Learning
Source: Adv Sci (Weinh). 2024 Jul 3;11(33):2307185. doi: 10.1002/advs.202307185 (PMC11434110; doi:10.1002/advs.202307185)
Supplement: Supplementary file 1 — Supporting Information [file ADVS-11-2307185-s003.pdf]

ADVANCED SCIENCE
Open Access

Supporting Information

for *Adv. Sci.*, DOI 10.1002/advs.202307185

Identification of a Hippocampus-to-Zona Incerta Projection involved in Motor Learning

*Zhuo-Hang Zhang, Bo Wang, Yan Peng, Ya-Wei Xu, Chang-Hong Li, Ya-Lei Ning, Yan Zhao, Fa-Bo Shan, Bo Zhang, Nan Yang, Jing Zhang, Xing Chen, Ren-Ping Xiong, Yuan-Guo Zhou and Ping Li**

Identification of a Hippocampus-to-Zona Incerta Projection involved in Motor Learning

Zhuo-Hang Zhang, *et al*.

*Corresponding author. E-mail:
ping__ping0074@sina.com;liping123123@tmmu.edu.cn

Supplemental figure 1

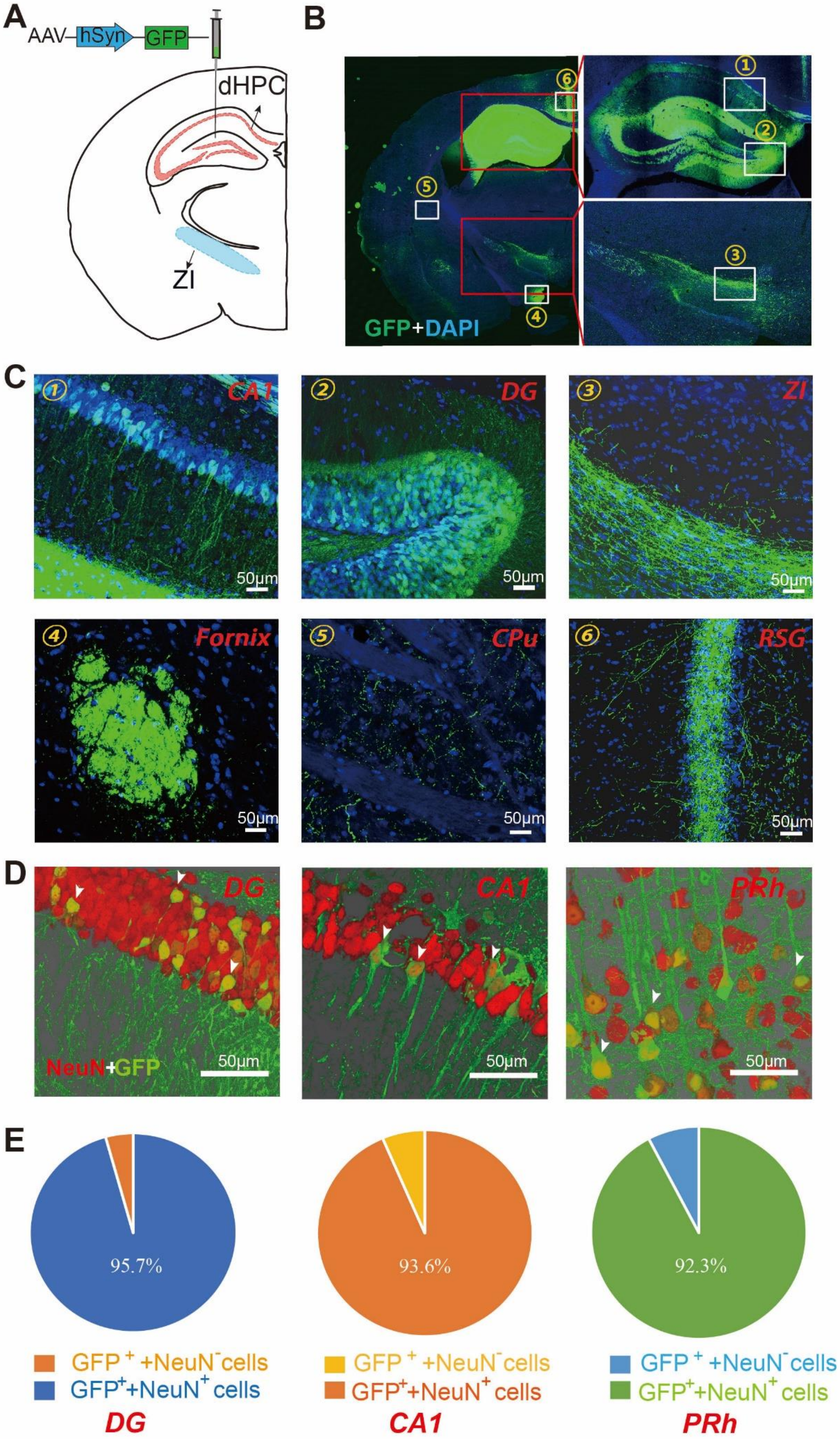

Figure S1 dHPC neurons project to the ZI.

(A) Schematic showing microinjections of AAV-hSyn-GFP into the dorsal hippocampus (dHPC) of mice. (B) Representative fluorescence signals in the mouse brain (*left*), dHPC (*right, upper*), and ZI (*right, lower*). (C) Higher magnification images of the sections in the white squares in (a, *right*). Green fluorescent-labelled cell bodies are observed in the DG and CA1, whereas green fluorescent-labelled fibres appear in the ZI, fornix, CPu, and RGS. (D) Representative immunohistochemical images of GFP (green) and NeuN+ (red) cells in the DG, CA1 and PRh taken from mice 1 month after microinjection with AAV2-retro-hSyn-eGFP. The white arrow indicates the colocalization of green and red fluorescent-labelled cells. (E) Pie chart of the percentage of GFP^{+}+ $NeuN^{+}$ cells or GFP^{+} + $NeuN^{-}$ cells to GFP-positive cells projecting to the ZI in the DG, CA1 and PRh. CPu, caudate putamen; RSG, granular retrosplenial cortex; PRh, perirhinal cortex.

Supplemental figure 2

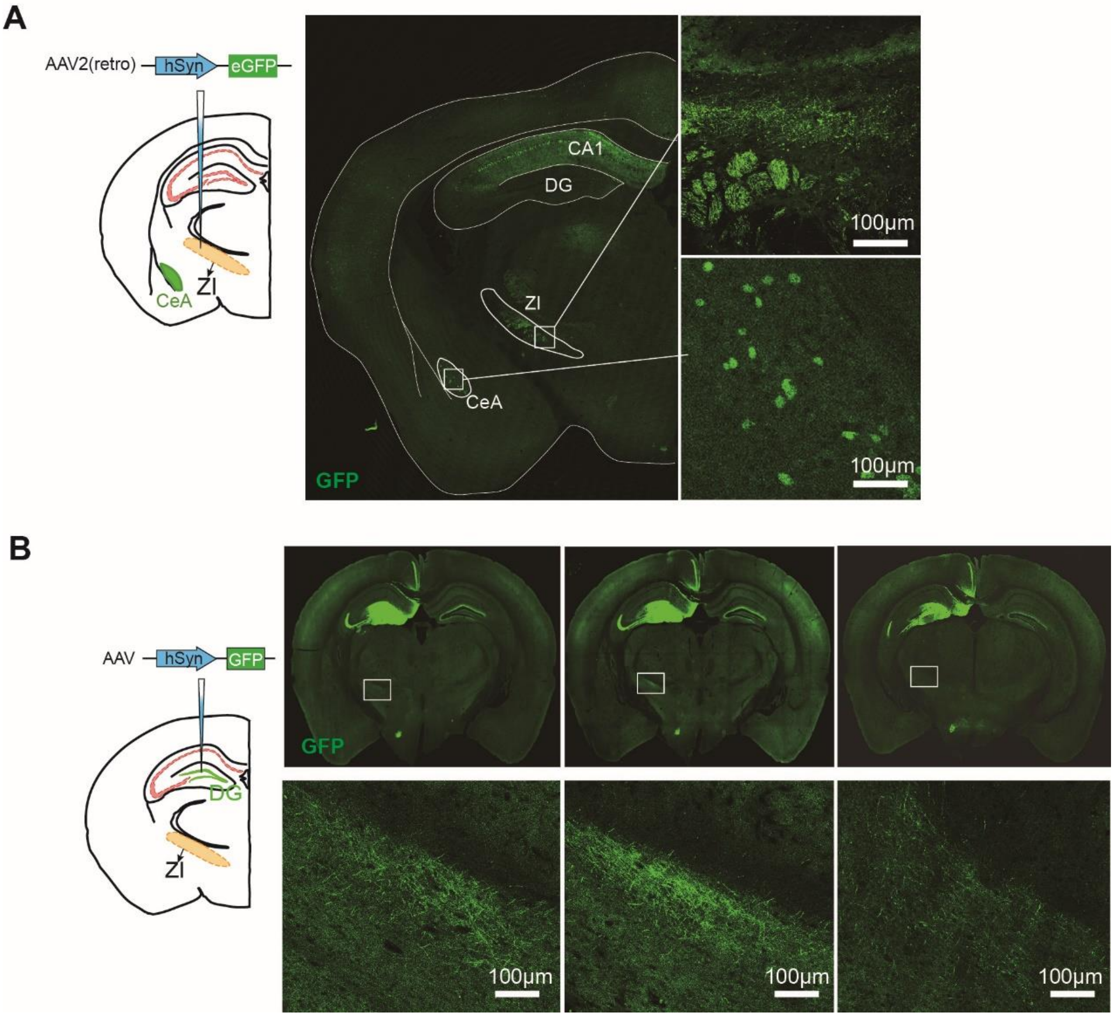

Figure S2 Projections from the central amygdala and dorsal DG to the ZI

(A) Schematic showing the viral labelling strategy, which involved microinjections of AAV2 (retro)-hSyn-GFP into the ZI in mice (*left*) and representative immunofluorescence images of GFP expression in the mouse brain (*middle*), ZI and central amygdala (*right,* higher magnification images of the sections in the white squares shown in the *middle*) 1 month after microinjection of AAV2 (retro)-hSyn-GFP into the ZI. (B) Schematic showing the viral labelling strategy using microinjections of AAV-hSyn-GFP into the dorsal DG in mice (*left*) and representative immunofluorescence images of GFP expression in the mouse brain (*right top*) and ZI (*right bottom,* higher magnification images of the sections in white squares in *right top*) 1 month after microinjection of AAV-hSyn-GFP into the dorsal DG.

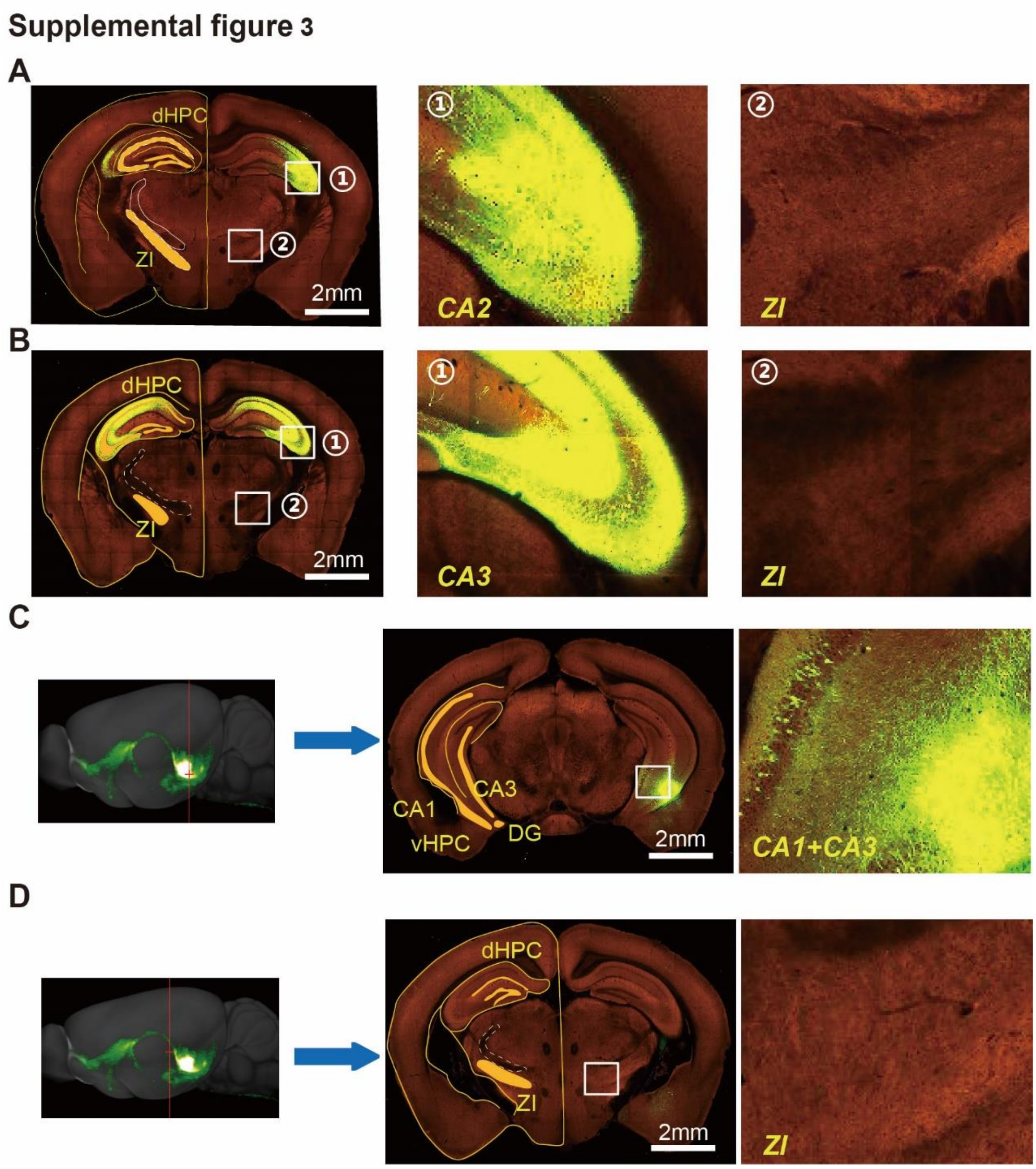

Figure S3 ZI neurons did not receive input from the dorsal CA2, dorsal CA3, ventral CA1 or ventral CA3

Panels from the Allen Reference Atlas (mouse.brain-map.org and atlas.brain-map.org). (A) Representative fluorescence images of GFP expression in mouse brain (*left)* or dorsal CA2 (*middle*) and ZI (*right*) through Cre-expressing AAV tracing of axonal projections. (B) Representative fluorescence images of GFP expression in the mouse brain (*left)* or dorsal CA3 (*middle*) and ZI (*right*) through Cre-expressing AAV tracing of axonal projections. Representative fluorescence images of GFP expression in the mouse brain (*middle*) and ventral HPC (vHPC) (*right*) (C) and in the mouse

brain (*middle*) and ZI (*right*) (D) through Cre-expressing AAV tracing of axonal projections; 3D thumbnails (*left*) reveal brain-wide projection patterns of green fluorescent-labelled fibres.

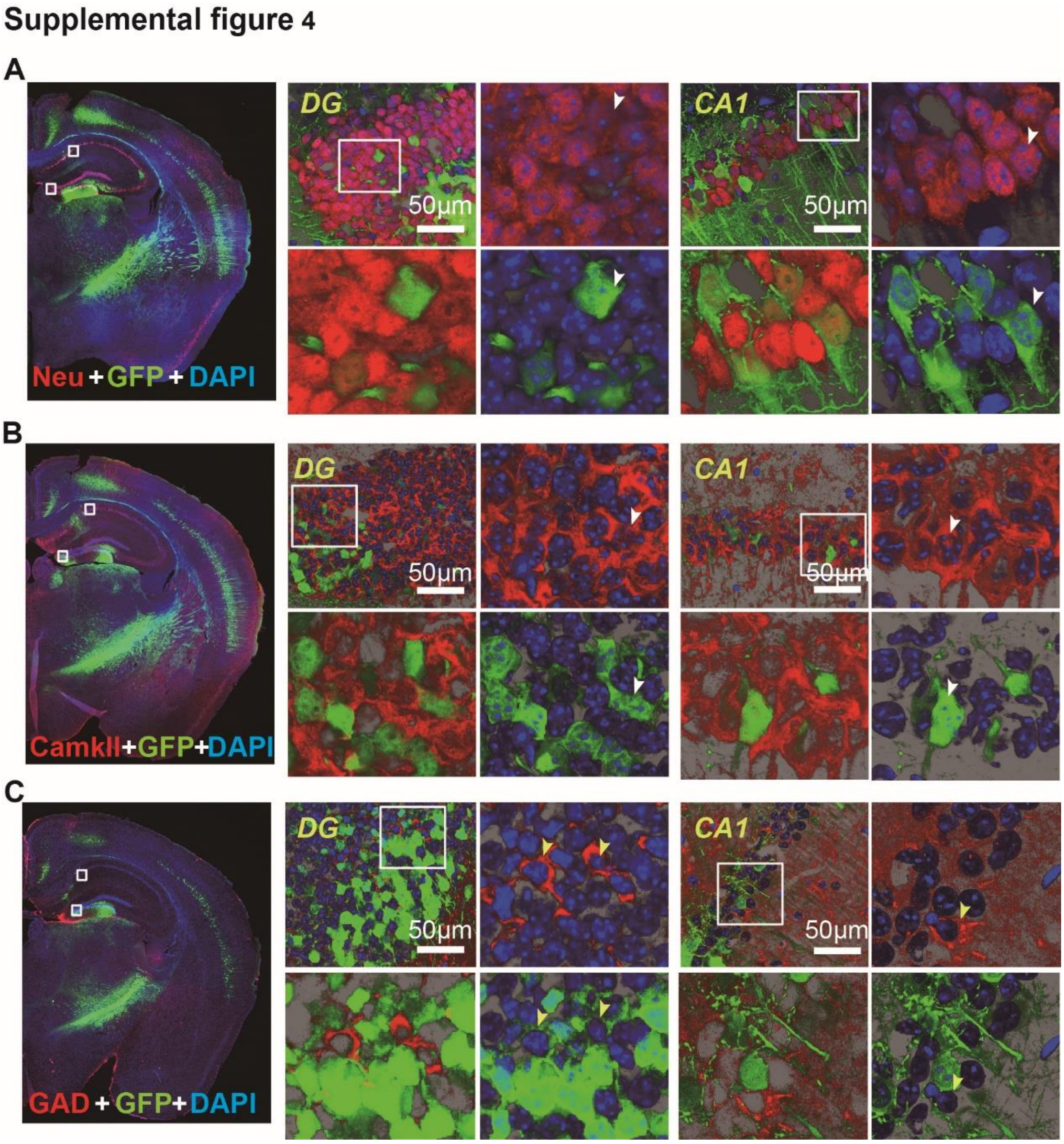

Figure S4 Projections from the dHPC to the ZI consist of excitatory neurons

Representative immunohistochemical images of eGFP-expressing (green) and NeuN+ (red) (A), eGFP-expressing (green) and CaMKII + (red) (B), and eGFP-expressing (green) and GAD + (red) (C) cells in the brain (*left*), DG and CA1 (*right*, higher magnification images of the sections in the white squares on the *left*) taken from mice microinjected with AAV2-retro-hSyn-eGFP after 1 month. The white arrow indicates the colocalization of green and red fluorescent-labelled cells, whereas the yellow arrow indicates the lack of colocalization of green and red fluorescent-labelled cells.

Supplemental figure 5

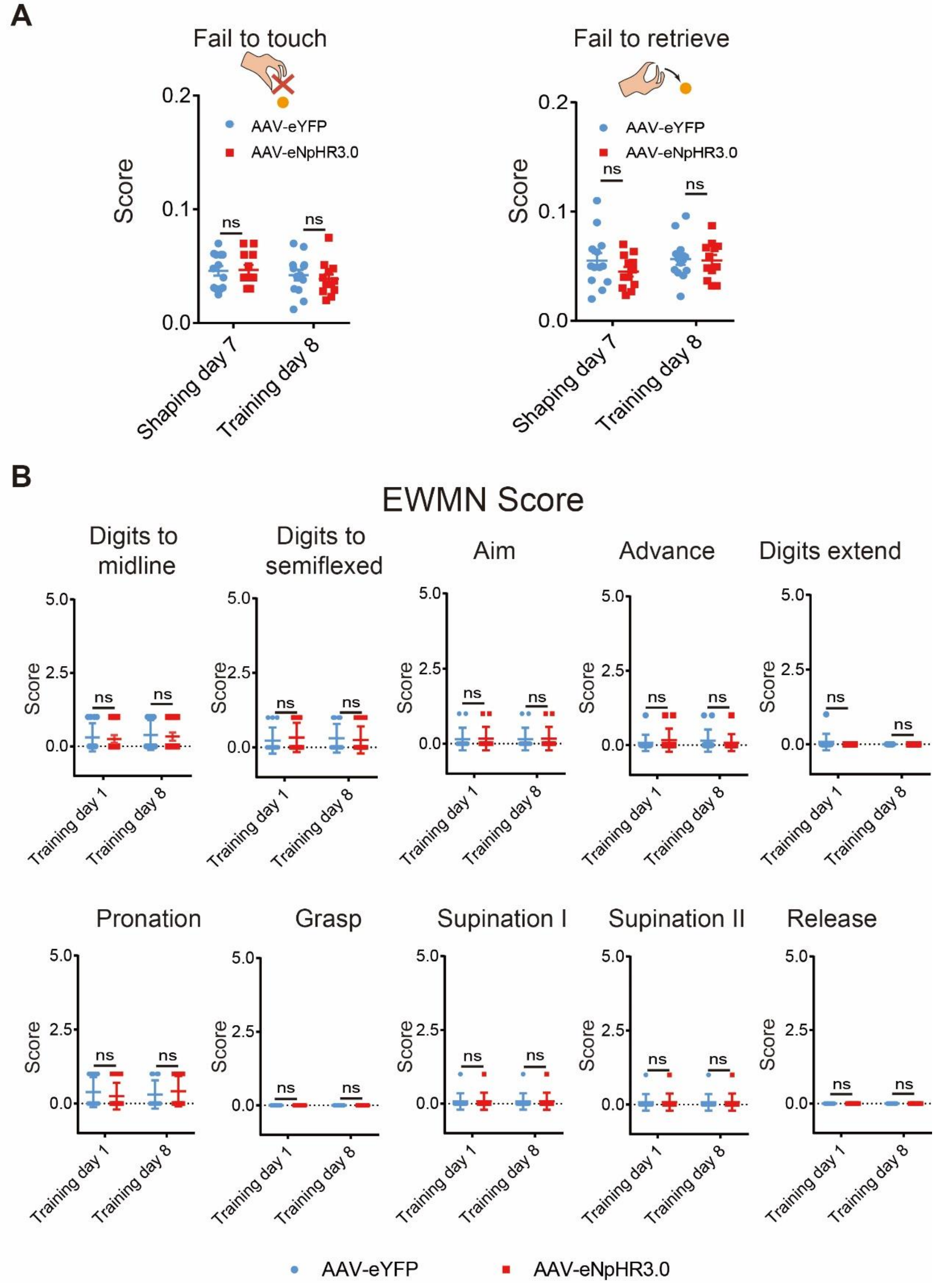

Figure S5 Optogenetic inhibition of projections from the dorsal HPC to the ZI had no effect on mouse digit dexterity.

(A) Rates of failure to touch and/or retrieve the pellet during the pellet reaching task after optogenetic inhibition of the projections from the dorsal HPC to the ZI in the AAV-eYFP and AAV-eNpHR3.0 mice. Two-tailed unpaired t tests were used to compare two groups (AAV-eYFP: n=13 animals vs. AAV-eNpHR3.0: n=12 animals: p=0.903 (failure to touch, training day 7), p=0.591 (failure to touch, training day 8), p=0.237 (failure to retrieve, training day 7), p=0.870 (failure to retrieve, training day 8)). The graph shows the mean ± SD. (B) EWMN scores for each of the 10 components of the pellet reaching movement after optogenetic inhibition of projections from the dorsal HPC to the ZI in AAV-eYFP and AAV-eNpHR3.0 mice, including digits to midline, digits semiflexed, aiming, advancement, digits extended, pronation, grasping, supination and release reflex. Two-tailed unpaired *t* tests were used (AAV-eYFP: n=13 animals vs. AAV-eNpHR3.0: n=12 animals; p=0.760 (digits to midline, day 1), p=0.800 (digits to midline, day 8), p=0.934 (digits semiflexed, day 1), p=0.934 (digits semiflexed, day 8), p=0.587 (aiming, day 1), p=0.760 (aiming, day 8), p=0.511 (advancement, day 1), p=0.606 (advancement, day 8), p=0.347 (digits extended, day 1), p=0.492 (pronation, day 1), p=0. 589 (pronation, day 8), p=0.955 (supination I, day 1), p=0.955 (supination I, day 8), p=0.955 (supination II, day 1) and p=0.955 (supination II, day 8)). The graph shows the mean ± SD.

Supplemental figure 6

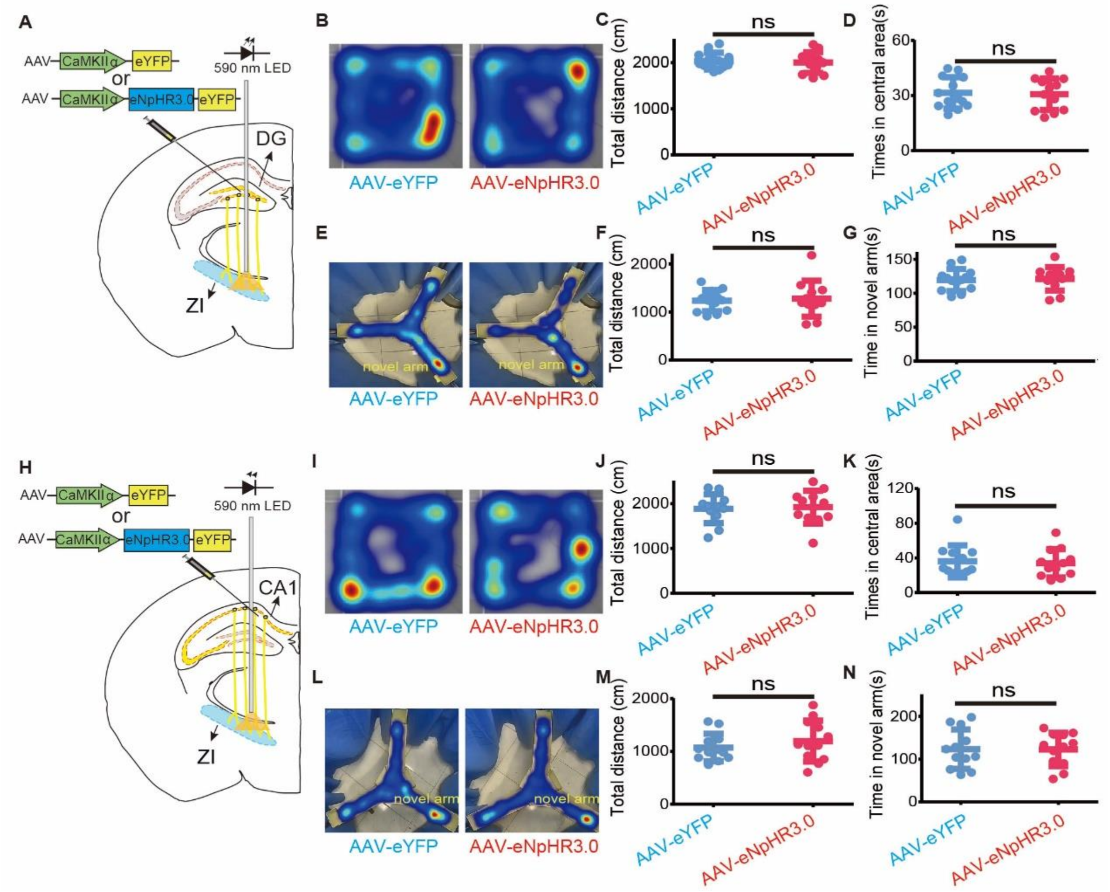

Figure S6 Optogenetic inhibition of projections from the dorsal DG or CA1 to the ZI had no effect on locomotor behaviour, anxiety-like behaviour or spatial memory.

(A) Schematic of the viral expression strategy and optogenetic approach for the inactivation of the terminals of the dorsal DG projections to the ZI. Representative moving track (B) and the total distance travelled (C) and time spent in the central area (D) in the open-field test for the two groups in which light was used to optogenetically inhibit projections from the dorsal DG to the ZI. Two-tailed unpaired t tests were used (AAV-eYFP: n=13 animals vs. AAV-eNpHR3.0: n=12 animals; *p*=0.487 and *p*=0.978 for the total distance travelled and time spent in the central area, respectively). Representative moving track (E), total distance travelled (F) and time spent in the novel arm (G) in the Y-maze test for the two groups in which light was used to optogenetically inhibit projections from the dorsal DG to the ZI in the test phase. Two-tailed unpaired t tests were used (AAV-eYFP: n=13 animals vs. AAV-

eNpHR3.0: n=12 animals; p=0.078 and p=0.824 for the total distance travelled and time spent in the novel arm, respectively. (H) Schematic of the viral expression strategy and optogenetic approach for the inactivation of the terminals of the dorsal CA1 projections to the ZI. Representative moving track (I) and the total distance travelled (J) and time spent in the central area (K) in the open-field test for the two groups stimulated by light to optogenetically inhibit projections from dorsal CA1 to the ZI. Two-tailed unpaired t tests were used (AAV-eYFP: n=12 animals vs. AAV-eNpHR3.0: n=12 animals; p=0.676 and p=0.693 for the total distance travelled and time spent in the central area, respectively. Representative moving track (L) and the total distance travelled (M) and time spent in the novel arm (N) in the Y-maze test for two groups stimulated by light to optogenetically inhibit projections from the dorsal CA1 to the ZI in the test phase. Two-tailed unpaired t tests were performed (AAV-eYFP: n=12 animals vs. AAV-eNpHR3.0: n=12 animals; p=0.207 and p=0.580 for the total distance travelled and time spent in the novel arm. The graph shows the mean ± SD. $^*p < 0.05$ was considered significant.

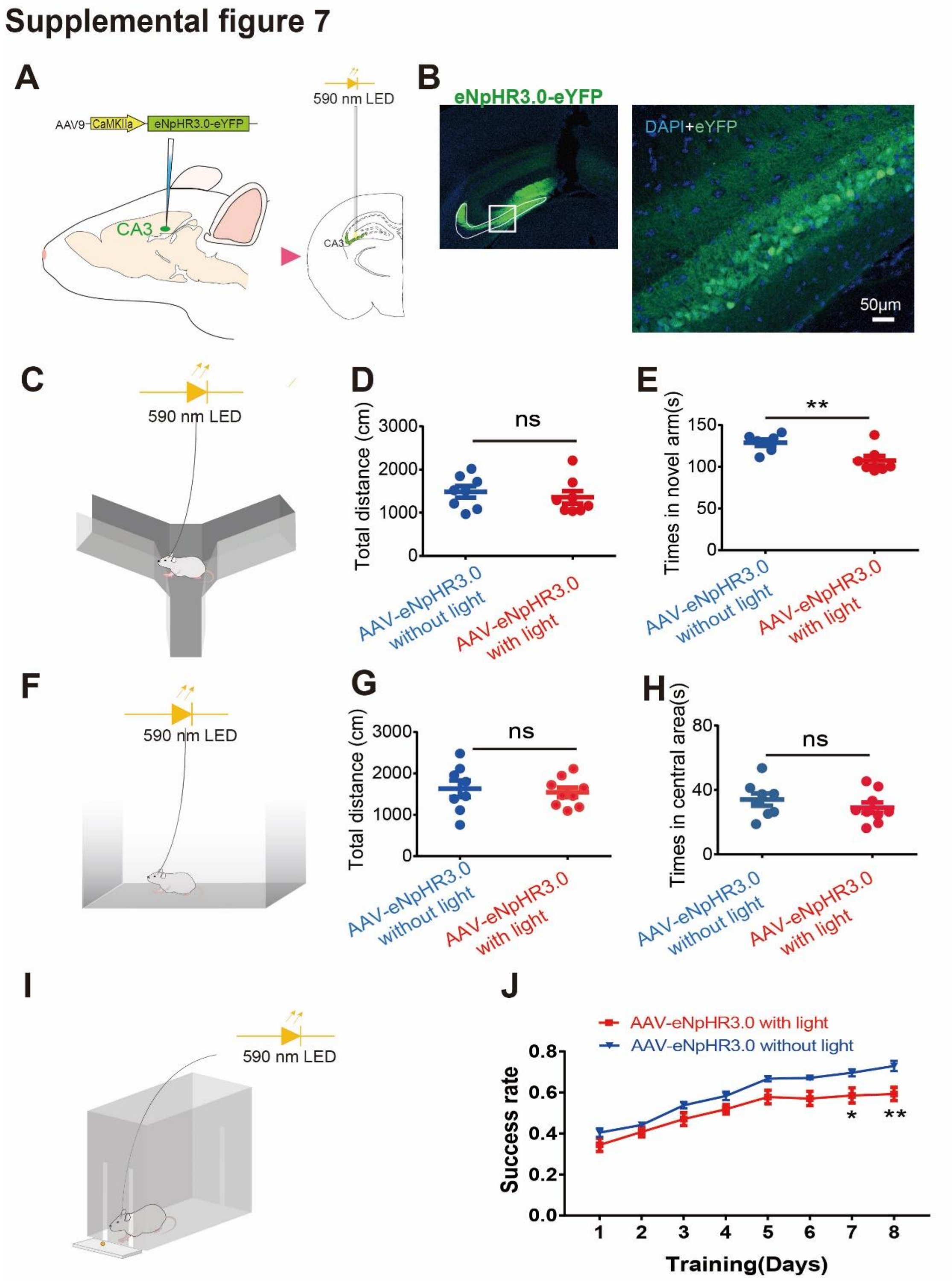

Figure S7. Behavioural changes after optogenetic inhibition of the CA3 of the dHPC. (A) Schematic of the viral expression strategy and optogenetic approach for inactivation of CA3 neurons in the dHPC. (B) Representative image of eYFP-

expressing neurons in the dHPC (*left*) and CA3 (*right*). (C) Schematic of the Y-maze test. The total distance travelled (D) and time spent in novel arms (E) in the Y-maze test for the two groups with or without light stimulation (eNpHR3.0+light: n =8 animals vs. eNpHR3.0–light: n = 8 animals; p=0.531 and p=0.008 for the total distance travelled and the time spent in the novel arm, respectively, according to two-tailed unpaired t tests). (F) Schematic of the open-field test. The total distance travelled (G) and time spent in the central area (H) in the open-field test for the two groups with or without light stimulation (eNpHR3.0+light: n =9 animals vs. eNpHR3.0–light: n =8 animals; p=0.694 and p=0.330 for the total distance travelled and time spent in the central area, respectively, according to two-tailed unpaired t tests). (I) Schematic of the single-pellet reaching test. (J) Changes in the success rate during the training phase for the two groups with or without light stimulation (eNpHR3.0+light: n =7 animals, eNpHR3.0–light: n = 8 animals). Two-way ANOVA was used, followed by Bonferroni's multiple comparisons test. $F(7, 120) = 32.97$, p<0.001 for the two groups (eNpHR3.0+light: n =9 animals, eNpHR3.0–light: n = 8 animals; p=0.878, 0.999, 0.584, 0.663, 0.145, 0.0618, 0.027, and 0.002 for days 1-8, respectively).

Supplemental figure 8

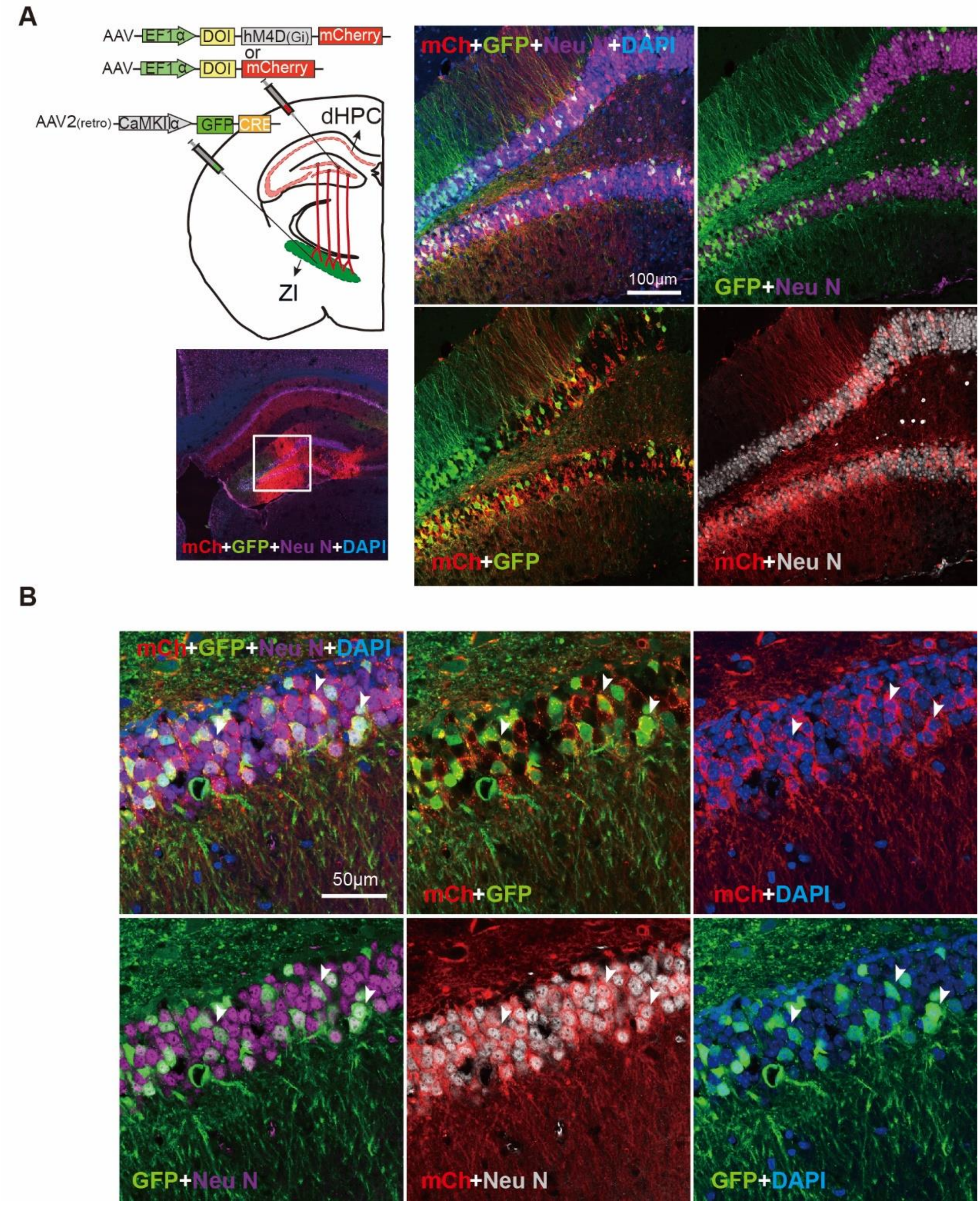

Figure S8 Expression of hM4D(Gi) on projection neurons of the dHPC-to-ZI projection.

(A) Schematic of the viral Cre/LoxP strategy for the expression of hM4D(Gi) on the neurons of the dorsal DG to ZI projection (*upper left*) and representative immunohistochemistry images of GFP-expressing (green), mCherry-expressing (red) and NeuN+ (purple or white) cells in the DG taken from mice 1 month after

microinjection with AAV (*lower left*). Higher magnification images of the sections in the white squares in the *lower left* (*right*). (B) Higher magnification images of (A). The white arrow indicates the colocalization of green, red and purple or white fluorescent-labelled cells.

Supplemental figure 9

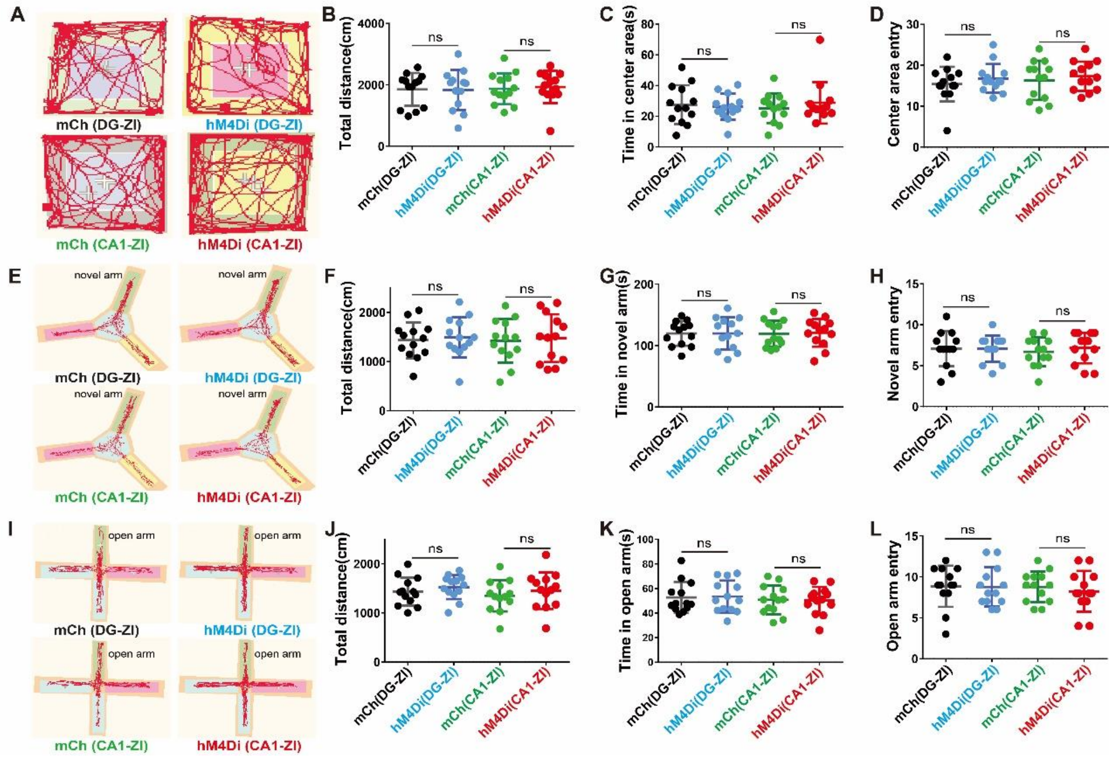

Figure S9 Chemogenetic inhibition of projections from the dorsal DG or CA1 to the ZI had no effect on locomotor behaviour, spatial memory or anxiety-like behaviour.

Representative moving track (A) and the total distance travelled (B), time spent in the central area (C) and number of central arm entries (D) in the open-field test in the different groups 30 min post-CNO injection. Two-tailed unpaired t tests were used (n=13 animals for each group; p=0.942, p=0.749, and p=0.373 for hM4Di(DG-ZI) vs. mCh(DG-ZI) and p=0.775, p=0.441, and p=0.553 for hM4Di(CA1-ZI) vs. mCh(CA1-ZI) for the total distance travelled, time spent in the central area and number of central arm entries, respectively). Representative moving track (E) and the total distance travelled (F), time spent in the novel arm (G) and number of novel arm entries (H) in the Y-maze test for the different groups 30 min after injection of CNO. Two-tailed unpaired t tests were used (n=13 animals for each group; p=0.692, p=0.999, and p=0.999 for hM4Di(DG-ZI) vs. mCh(DG-ZI) and p=0.769, p=0.867, and p=0.521 for hM4Di(CA1-ZI) vs. mCh(CA1-ZI) in the total distance travelled, times in the novel arm and number of novel arm entries, respectively). Representative moving track (I)

and the total distance travelled (J), time spent in the open arm (K) and number of open arm entries (L) in the plus-maze test for the different groups 30 min post-CNO injection. Two-tailed unpaired t tests were used (n =13 animals for each group; p=0.376, p=0.887, and p=0.937 for hM4Di(DG-ZI) vs. mCh(DG-ZI) and p=0.451, p=0.902, and p=0.539 for hM4Di(CA1-ZI) vs. mCh(CA1-ZI) in the total distance travelled, time spent in the open arm and the number of open arm entries, respectively). The graph shows the mean ± SD.

Movie S1 3D confocal laser scanning microscopy (CLSM) reconstruction of green fluorescent-labelled cell bodies in the dorsal DG

Representative 3D-reconstructed green fluorescent-labelled cell bodies in the dorsal DG 1 month after microinjection of AAV2-retro-hSyn-eGFP into the ZI of mice. The nuclei are stained blue with DAPI.

Movie S2 3D CLSM reconstruction of green fluorescent-labelled cell bodies in the dorsal CA1

Representative 3D-reconstructed green fluorescent-labelled cell bodies in the dorsal CA1 1 month after microinjection of AAV2-retro-hSyn-eGFP into the ZI of mice. The nuclei are stained blue with DAPI.

Movie S3 3D CLSM reconstruction of dorsal CA1 terminals in the ZI

Representative 3D-reconstructed green fluorescent-labelled terminals of neurons projecting from the dorsal CA1 to the ZI in the mouse brain 1 month after microinjection of AAV-hSyn-GFP into the dorsal CA1. The nuclei are stained blue with DAPI.
